# Xer Recombinase and Genome Integrity in *Helicobacter pylori*, a Pathogen without Topoisomerase IV

**DOI:** 10.1371/journal.pone.0033310

**Published:** 2012-04-12

**Authors:** Aleksandra W. Debowski, Christophe Carnoy, Phebe Verbrugghe, Hans-Olof Nilsson, Jonathan C. Gauntlett, Alma Fulurija, Tania Camilleri, Douglas E. Berg, Barry J. Marshall, Mohammed Benghezal

**Affiliations:** 1 Ondek Pty Ltd and Helicobacter pylori Research Laboratory, School of Pathology & Laboratory Medicine, M504, Marshall Centre for Infectious Disease Research and Training, University of Western Australia, Nedlands, Washington,; 2 United States of America Center for Infection and Immunity of Lille, INSERM U 1019, CNRS UMR 8204, Univ Lille Nord de France, Institut Pasteur de Lille, Lille, France; 3 Department of Molecular Microbiology, Washington University School of Medicine, St. Louis, Missouri, United States of America; University of Hyderabad, India

## Abstract

In the model organism *E. coli*, recombination mediated by the related XerC and XerD recombinases complexed with the FtsK translocase at specialized *dif* sites, resolves dimeric chromosomes into free monomers to allow efficient chromosome segregation at cell division. Computational genome analysis of *Helicobacter pylori*, a slow growing gastric pathogen, identified just one chromosomal *xer* gene (*xerH*) and its cognate *dif* site (*difH*). Here we show that recombination between directly repeated *difH* sites requires XerH, FtsK but not XerT, the TnPZ transposon associated recombinase. *xerH* inactivation was not lethal, but resulted in increased DNA per cell, suggesting defective chromosome segregation. The *xerH* mutant also failed to colonize mice, and was more susceptible to UV and ciprofloxacin, which induce DNA breakage, and thereby recombination and chromosome dimer formation. *xerH* inactivation and overexpression each led to a DNA segregation defect, suggesting a role for Xer recombination in regulation of replication. In addition to chromosome dimer resolution and based on the absence of genes for topoisomerase IV (*parC*, *parE*) in *H. pylori*, we speculate that XerH may contribute to chromosome decatenation, although possible involvement of *H. pylori*'s DNA gyrase and topoisomerase III homologue are also considered. Further analyses of this system should contribute to general understanding of and possibly therapy development for *H. pylori*, which causes peptic ulcers and gastric cancer; for the closely related, diarrheagenic Campylobacter species; and for unrelated slow growing pathogens that lack topoisomerase IV, such as *Mycobacterium tuberculosis*.

## Introduction

Crossovers between circular monomeric chromosomes generate dimers and interlocked (catenated) structures that cannot segregate properly at cell division [1]. Bacteria with circular chromosomes generally contain site-specific tyrosine Xer recombinases that act at cognate *dif* sites near where replication terminates and that resolve chromosome dimers to free monomers [1], [2]. Deletion of *dif* or inactivation of a *xer* recombinase gene causes formation of abnormally partitioned nucleoids and cell filamentation in *E. coli* type model organisms [3]. Cell filamentation results from SulA-mediated inhibition of cell division, is induced in the SOS response to DNA and chromosome breakage [4]. Chromosome dimer resolution in most bacterial species, including *E. coli*, is mediated by two related recombinases, XerC and XerD, that function as a pair of heterodimers and that target a 28-bp *dif* site. The *dif* site is presented to the Xer complex by the FtsK DNA translocase protein. FtsK is anchored at incipient cell division septa, and interacts with a set of oriented and highly repeated 8 bp named KOPS sequences (FtsK-orienting polar sequences). The *E. coli* chromosome's *dif* region is rich in KOPS sites, which are in opposite orientation on each side of the *dif* site. Orientation-specific KOPS recognition and asymmetry in the KOPS distribution direct FtsK-chromosome interactions to effectively guide presentation of *dif* to the XerC-XerD complex for recombination. FtsK interacts specifically with the XerD component of the XerCD complex and with several other proteins including Topoisomerase IV, which are likely to be important for efficient, well-regulated chromosome separation and segregation [2], [5], [6]. Many thousands of topological links arise as circular chromosomal DNAs are unwound during replication [7]. In *E. coli* interlocked (catenated) chromosomes are resolved efficiently to monomers by topoisomerase IV [8], which is essential [9], primarily because of its high capacity to resolve interlocked chromosomes, and thereby allow efficient chromosome segregation, apace with rapid cell division [8]. Of note, DNA gyrase (responsible for DNA supercoiling) and Xer recombination may play secondary roles in decatenation [7], [8],[10]. This is illustrated by the ability of *E. coli*'s XerCD-*dif*-FtsK system can substitute for topoisomerase IV [11] to remove catenane links between circular DNAs *in vitro* without topoisomerase IV [11]; and the suppression of a temperature sensitive (conditional lethal) topoisomerase IV mutation by XerCD-*dif* recombination in *E. coli* producing an engineered FtsK protein with no septum anchor (FtsK_50C_) that is thus soluble in the cytoplasm [7]. This Xer-mediated resolution of catenated DNAs entails multiple interconversions of catenated monomers and knotted dimers, removing a link at each step.

Xer/*dif* recombination systems have been detected computationally in many phyla [12], [13] including Proteobacteria [14], Firmicutes [15], [16] and Archae [17], [18]. Our *in silico* analyses revealed that more than 85% proteobacterial species contain a conventional *E. coli*-type system in which related XerC and XerD recombinases act as heterodimers on cognate *dif* sites, with each Xer protein having a distinct role. However, *Helicobacter pylori*, the gastric pathogen implicated in peptic ulcer disease and gastric cancer, was inferred to contain just a single Xer recombinase, which was named XerH, as do the related *Campylobacters*, which cause diarrheal disease, and all other members of *H. pylori*'s epsilon subgroup of Gram negative proteobacteria [14]. Single Xer recombinase systems were also found in the Gram positive Streptococci and Lactococci (XerS) [15] and in Archaea (XerA) [17], [18]. *ftsK* homologues are found in nearly all eubacterial species including the epsilon proteobacteria and Streptococci and Lactococci. Interestingly, several slow growing bacterial pathogenic genera, including *Helicobacter*, *Campylobacter* and *Mycobacterium*, lack *parC* and *parE* topoisomerase IV subunit genes [19]. Further complicating inferences about XerH action in the case of *H. pylori*, many strains contain a second divergent *xer* recombinase gene, *xerT*, generally within a large TnPZ transposon [20], [21]. Although its encoded XerT protein is needed for transposon excision and conjugative transfer, and probably also functions as a transposase, the possibility of XerT collaborating with XerH for chromosome resolution (as with XerC and XerD in *E. coli*) also merited testing.

The experiments presented here demonstrate site-specific recombination at *H. pylori difH* sites, and show that it requires XerH, FtsK and an intact *difH* sequence, but not XerT, and bring into focus the need to learn how catenanes are processed in the many other slow growing human pathogens that, like *H. pylori*, lack topoisomerase IV.

## Materials and Methods

### Bacterial strains and culture conditions

The *H. pylori* strains and plasmids used in this study are listed in Table S1. Streptomycin resistant *rpsL*-mutant strains were used for transformation with the *difH* repeat (*rpsL*-*cat* containing) cassette [22], [23]. *H. pylori* strains were routinely grown at 37°C under microaerobic conditions on Columbia blood agar (CBA) plates containing 5% horse blood and Dent's antibiotic supplement (Oxoid). When appropriate, antibiotic selection in *H. pylori* was carried out by supplementing media with chloramphenicol, streptomycin, and/or kanamycin at final concentrations of 10 µg/ml. *Escherichia coli* DH5α was grown in Luria-Bertani broth. When necessary, antibiotics were added to the following final concentrations: ampicillin, 100 µg/ml; kanamycin, 50 µg/ml; and chloramphenicol, 20 µg/ml. *H. pylori* cultures were incubated at 37°C in sealed jars using the Anoxomat™ MarkII system (Mart Microbiology B.V., The Netherlands) after one atmosphere replacement using the following gas composition N2∶H2∶CO2, 85∶5∶10.

### Oligonucleotides

The oligonucleotides used in this study are listed in Table S2

### Assays of *difH* site recombination

**Figure 1:**
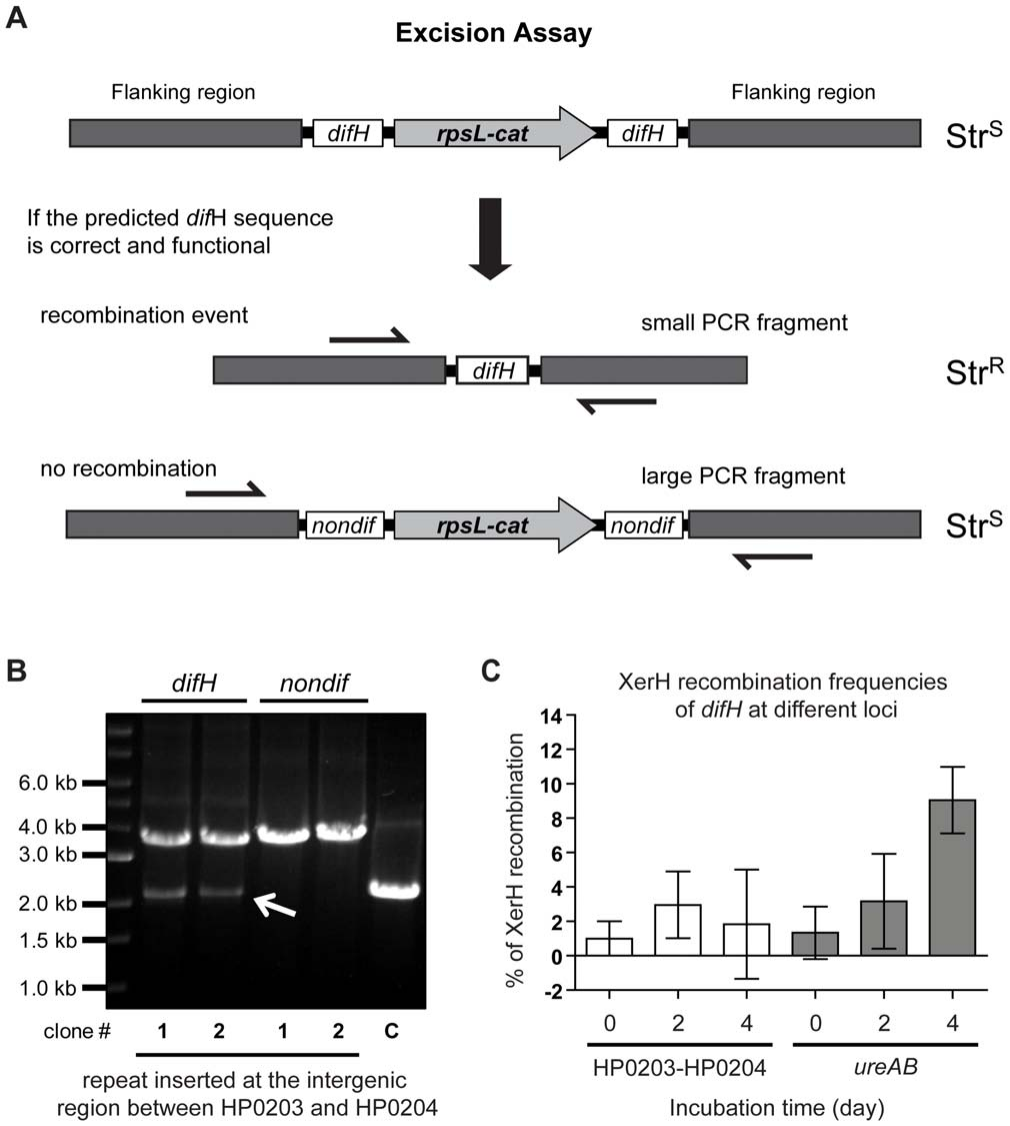
XerH/*difH* site-specific recombination assay in *Helicobacter pylori.* (A) Schematic depiction of XerH excision assay. The *difH* repeat cassette, consisting of streptomycin susceptibility-chloramphenicol resistance genes (*rpsL*-*cat*) flanked by *difH* sequence, was introduced into the *H. pylori* 26695^Str^ (WT) genome by natural transformation and homologous recombination between genes HP0203 and HP0204 or in place of genes *ureA* and *ureB*. Recombination at *difH* sites leads to excision of *rpsL*-*cat* and one *difH* sequence, detectable by a PCR fragment 1.5 kb smaller than that from parental *difH* repeat cassette containing DNA. A cassette with *nondif* sequence flanking *rpsL-cat* served as a negative control. (B) Results of *difH* recombination assay in *H. pylori* 26695^Str^ (WT) with 40 bp *difH* direct repeats or 40 bp *nondif* DNA direct repeats at the HP0203-HP0204 locus. With each of these cassettes, two independent clones were tested by diagnostic PCR. The 2.1 kb and 3.6 kb PCR products come *difH* recombinant and parental (*difH* repeat containing) DNAs, respectively. Lane C, control from wild-type *H. pylori* without *difH* repeat cassette. (C) *difH* recombination frequencies for *difH* repeat cassette located at HP0203-HP0204 or *ureAB* loci. Cells were grown on non-selective media for two or four days, re-streaked for single colonies, and ∼100–200 colonies were tested for retention or loss of *rpsL* and *cat* genes by replica plating to streptomycin and to chloramphenicol containing media. Experiments were performed in triplicates; horizontal bars indicate means and standard deviation.

To test if the putative 40-bp *H. pylori dif* sequence (*difH*) ATTTAAAAGTTTGAAAAGTGCAGTTTTCATAACTAAATGA) was functional, a recombination assay was developed using a cassette containing both selectable (*cat*) and counterselectable (*rpsL*) genes, *rpsL-cat* (streptomycin susceptibility, chloramphenicol resistance, respectively), flanked by *difH* sites [22], [24], [25]. This cassette was generated by PCR amplification from genomic DNA containing *rpsL-cat* using primers DifHPF and DifHPR, which contain direct repeats of *difH* and *Bam*HI restriction sites near their 5′ ends. A control cassette containing 40 bp of sequence unrelated to *difH* was generated with primers NondifF and NondifR. These PCR products were cloned as described [26] to create plasmids pHInt_difH-RCAT-difH and pHInt_nondif-RCAT-nondif. Constructs were sequenced using primers SEQdifF and SEQdifR to ensure that *difH* and ‘*nondif*’ sequences were intact. Natural transformation of a derivative of *H. pylori* strain 26695 made resistant to streptomycin by mutation in its normal *rspL* gene (called 26695^Str^) with pHInt_difH-RCAT-difH and pHInt_nondif-RCAT- nondif [26] was used to place these cassettes in the *H. pylori* chromosome between genes HP0203 and HP0204 (strains 26695^Str^ *HP0203-4*::*difH*-RCAT and 26695^Str^ *HP0203-4*::*nondif*-RCAT, respectively). Chromosomal DNAs from the resulting chloramphenicol-resistant transformants were checked for streptomycin sensitivity (which is dominant to resistance in *rpsL* ^mut^/*rpsL* ^WT^ partial diploids). These transformants and their descendants were also checked for correct insertion of the *difH* repeat cassette and for recombination at *difH* sites by PCR using primers 0203F and 0204R. Additionally, chloramphenicol-resistant transformants were subcultured on streptomycin-containing agar when needed to select for or to quantify rates of loss of the cassette. Chromosomal DNAs of representative streptomycin-resistant, chloramphenicol-sensitive derivatives were sequenced to confirm recombination at *difH* sites as diagrammed in Figure 1.

Due to the low *difH* recombination rates in the HP0203-HP0204 intergenic region, the *difH* repeat cassette was also placed at the *ureAB* locus for further studies. Synthetic sequences containing direct repeats of wild-type or mutant *difH* sites separated by a *Bgl*II restriction site and flanked with *Bam*HI sites were synthesized and cloned into plasmid pMA-RQ from Geneart (AG Regensburg, Germany) (mutant *difH* sequences shown in Table S1). A *Bam*HI fragment containing the *rpsL-cat* cassette was cloned into *Bgl*II digested plasmids pDifWT, pDifM1, pDifM2, and pDifM3 (Table S1) to give *rpsL-cat* flanked with WT or mutated *difH* sequences in pDifWT-RC, pDifM1-RC, pDifM2-RC, pDifM3-RC respectively (Table S1). These *difH* flanked DNAs were excised with *Bam*HI and cloned into plasmid pUreAB [27], a pBluescript-derived plasmid containing regions of homology for chromosomal replacement of [or insertion between] the *ureA* and *ureB* (urease) genes. This resulted in plasmids pUreAB_DifWT-RC, pUreAB_DifM1-RC, pUreAB_DifM2-RC and pUreAB_DifM3-RC, which were used for transformation of *H. pylori* strain 26695^Str^ to create 26695^Str^ *ureAB*::*difH*WT-RC, *ureAB*::*difH*M1-RC, *ureAB*::*difH*M2-RC and *ureAB*::*difH*M3-RC. Using primers UreABF and UreABR, chromosomal DNA of the resulting chloramphenicol-resistant transformants was checked for the correct insertion of the *difH* repeat cassette and for *difH* recombination at the *ureAB* locus.

### *H. pylori* mutants used to assess *H. pylori xerH* and *xerT* roles in *difH* recombination

*xerH* is the only *xer* recombinase gene found in every *H. pylori* genome, although many strains, including 26695 (used here), also contain another *xer* family gene, *xerT* [21]. Strain 26695^Str^ derivatives with null mutations in these *xer* genes were constructed in order to test each gene's role in *dif* site recombination. *xerH::rpsL-cat* and *xerT::rpsL-cat* insertion mutant constructs were generated by PCR with overlapping primers [28], [29]. Briefly, for *xerH* (HP0675), DNAs flanking and including much of this gene were amplified from 26695 genomic DNA using primers XerHrcat1 and XerHrcat2, and XerHrcat3 and XerHrcat4, respectively; and the *rpsL-cat* cassette was amplified using primers XerHrcat5 and XerHrcat6. Nested primers XerHrcat7 and XerHrcat8 were used for splicing overlap extension (SOE) PCR to generate a DNA segment containing *rpsL-cat* inserted within the HP0675 ORF, which would be suitable for transformation. The same strategy, using primers XerTrcat1 through XerTrcat8, was used to generate *xerT::rpsL-cat* mutant of the *xerT* gene (HP0995). Natural transformation of *H. pylori* strain 26695^Str^ with these products yielded 26695^Str^ *xerH::rpsL-cat* and 26695^Str^ *xerT::rpsL-cat*. DNAs of transformants were checked for correct allelic insertion by PCR. Simple unmarked *xerH* and *xerT* deletion alleles (Δ*xerH* and Δ*xerT*) were then made by SOE PCR. Briefly, for *xerH*, 1-kb DNA segments flanking HP0675 were PCR amplified using primers XerHdel1 and XerHdel2, and XerHdel3 and XerHdel4, and fused in a second SOE PCR using nested primers XerHdel5 and XerHdel6. The Δ*xerT* allele was made similarly with primers XerTdel1 through XerTdel6. *H. pylori* strains containing *xerH::rpsL-cat* and *xerT::rpsL-cat* constructs were transformed with corresponding simple deletion DNAs, streptomycin resistance was selected, and transformants were screened for loss of chloramphenicol resistance and further checked by PCR. This resulted in strains 26695^Str^ Δ*xerH* and 26695^Str^ Δ*xerT*.

To assess the role of XerH and XerT in *difH* recombination, these Δ*xerH* and Δ*xerT* strains were transformed with pHInt_difH-RCAT-difH to give 26695^Str^ Δ*xerH*; *HP0203-4::difH-RCAT* and 26695^Str^ Δ*xerT*; *HP0203-4::difH-RCAT*; or with pUreAB_DifWT-RC to give 26695^Str^ Δ*xerH*; *ureAB::difHWT-RC* and 26695^Str^ Δ*xerT*; *ureAB::difHWT-RC* (*difH* flanking *rpsL-cat* at the *ureAB* locus). Chloramphenicol-resistant transformants were selected, and then assayed for *difH* site recombination by appearance of streptomycin resistance and loss of chloramphenicol resistance and by PCR.

### Complementation of Δ*xerH* mutation

The *xerH* (HP0675) ORF was amplified from 26695 genomic DNA using primers XerHF and XerHR and the product was cloned downstream of the strong *ureA* promoter of pTrpA-up (Table S1) using *Nde*I and *Sal*I restriction sites to create pTrpA-upXerH. Transformation of strain 26695^Str^ Δ*xerH* with pTrpA-RC yielded 26695^Str^ Δ*xerH*recip, which was in turn transformed with pTrpA-upXerH to create strain 26695^Str^ *xerH* comp, (Δ*xerH* complemented with highly expressed *xerH* gene at the chromosomal *trpA* locus, under *ureA* promoter control). Chromosomal DNAs of the resulting transformants were checked by PCR for correct allelic replacement at *trpA* and to verify that the cloned *xerH* gene was not at the normal chromosomal *xerH* locus. Two independently generated 26695^Str^ *xerH* comp clones were then transformed with pUreAB_DifWT-RC to give 26695^Str^ *xerH* comp; *ureAB::difHWT-RC* (*difH* flanking *rpsL-cat* at the *ureAB* locus). Chloramphenicol-resistant transformants were selected and assayed for recombination events at *difH* sites.

### Construction of *H. pylori* mutants used to assess susceptibility to DNA damage

SOE PCR was used to generate a *ruvC::rpsL-cat* construct. For *ruvC* (HP0877), 1-kb DNA segments flanking HP0877 were amplified from 26695 genomic DNA using primers RuvCrcat1 and RuvCrcat2, and RuvCrcat3 and RuvCrcat4 respectively; and the *rpsL-cat* cassette was amplified using primers RuvCrcat5 and RuvCrcat6. Nested primers RuvCrcat7 and RuvCrcat8 were used to generate a *ruvC::rpsL-cat* containing SOE PCR product. Natural transformation of the *H. pylori* strain 26695 with the final SOE PCR products was performed to create 26695^Str^ Δ*ruvC*.

make a *recG* knockout mutant, two 1-kb DNA fragments upstream and downstream of the HP1523 ORF were amplified using primers RecGkan1 and RecGkan2, and RecGkan3 and RecGkan4 and fused by SOE PCR using primers RecGkan1 and RecGkan4, creating unique *Eco*RI and *Bam*HI restriction sites. The final PCR product was blunt end cloned into pHSG576 [30] to give pHRecG. The *aphA* cassette, conferring kanamycin resistance (Kan^R^), was cloned into *Eco*RI and *Bam*HI digested pHRecG, creating a nonpolar replacement of *recG*, pHRecG-Km. This plasmid was used for 26695^Str^ transformation to create 26695^Str^ Δ*recG*.

Several attempts to construct the DNA segment to make a *recA* knockout by SOE PCR failed. Therefore, *in vitro* transposition was used to insert the *rpsL-cat* cassette into *recA*. The HP0153 ORF was amplified by PCR, using primers RecAF and RecAR, and cloned into pGEMT-Easy (Promega, Madison, WI) to create pRecA. *In vitro* transposition of a segment containing *rpsL-cat* flanked by ends of phage Mu into pRecA was done using MuA transposase (Finnzymes, Finland, F-750) to generate a library of pRecA-RC clones. *H. pylori* strain 26695^Str^ was transformed to chloramphenicol resistance using this plasmid library to generate 26695^Str^ *recA*::mu-*rpsL-cat*. Chromosomal DNA was isolated from transformants, and insertion of the mu-*rpsL-cat*-mu cassette into ORF HP0153 was confirmed by PCR. A similar strategy was used to truncate the *H. pylori ftsK* homologue. Primers FtsKF and FtsKR were used to amplify the HP1090 ORF and generate the *ftsK* containing plasmid pFtsK. *In vitro* transposition of mu-*rpsL-cat* into pFtsK generated a library of pFtsK-RC mutant DNAs which was used in transformation to make strain 26695 *ftsK*::mu-*rpsL-cat*. Transformants were characterised by PCR and DNA sequencing, and one with an insertion at the 454^th^ codon from *ftsK*'s 3′ end was identified.

Double and triple mutants were obtained by transforming 26695^Str^ Δ*xerH* with PCR products containing *ruvC::rpsL-cat* to create 26695^Str^ Δ*xerH*Δ*ruvC*; 26695^Str^ Δ*recG* with a *recA*::mu-*rpsL-cat*-containing PCR product to create 26695^Str^ Δ*recG recA*::mu-*rpsL-cat*; 26695^Str^ Δ*xerH*, 26695^Str^ *ruvC::rpsL-cat* and 26695^Str^ Δ*xerH*Δ*ruvC* with pHRecG-Km to give 26695^Str^ Δ*xerH*Δ*recG*, 26695^Str^ Δ*ruvC*Δ*recG* and 26695^Str^ Δ*xerH*Δ*ruvC*Δ*recG*.

### Construction of *xerH* and *ruvC* mutants in strain X47

To generate Δ*xerH* derivatives of X47 (which already contains an *rpsL* streptomycin resistance allele), this strain was transformed with genomic DNA from strain 26695^str^ *xerH::rpsL-cat*. Genomic DNA isolated from the resulting transformants was used as template for PCR to confirm *rpsL-cat* insertion at *xerH*. A pool of X47 *xerH::rpsL-cat* clones was transformed with the PCR product obtained from 26695^Str^ Δ*xerH* (unmarked deletion), with selection for streptomycin resistance and chloramphenicol susceptibility. Genomic DNA from the resulting X47 Δ*xerH* transformants was used as template for PCR to confirm clean deletion of *xerH*, as described for 26695.

To generate the *ruvC* mutant in X47 background, wild-type X47 was transformed with genomic DNA from strain 26695 Δ*ruvC::rpsL-cat*. Genomic DNA isolated from the resulting transformants was used as template for PCR to confirm *rpsL-cat* insertion at the *ruvC* locus as described for 26695.

### UV susceptibility assay

Fresh cultures of *H. pylori*, passaged the day before, were suspended in PBS (pH = 7.2) and standardized to an OD_600_ = 2. 50 µl aliquots of bacterial suspension was placed into a single well of a UV transparent 96 well plate (NUNC) and exposed to UV light at 312 nm using the TFX-35M transluminator (LifeTechnologies, Carlsbad, CA) at a distance of 45 cm for 0 to 75 sec. Serial dilutions of irradiated cells were plated onto CBA plates and incubated at 37°C for four days. Colonies were counted and percent survival was calculated. UV susceptibility experiments were repeated at least three times on two independent clones for each *H. pylori* strain.

### DNA content analysis by Fluorescence Activated Cell Sorting (FACS)

The DNA content analysis was performed as described [31]. Briefly, *H. pylori* cells were grown on blood agar plates and inoculated in BHI medium containing 10% Newborn Calf Serum (NCS) at OD_600_ = 2 and incubated in 100 µl in 96 well plate overnight (18 h) with shaking at 100 rpm. The bacteria were collected by centrifugation, the pellet was resuspended in 300 µl PBS and added to 900 µl PBS with 4% paraformaldehyde and samples were incubated 15 min at room temperature. The bacterial pellet was washed with PBS, resuspended in 100 µg/ml RNase A and 2 µg/ml Hoechst 333421 (Sigma) in PBS and incubated for 10 min at 37°C. The bacterial pellet was washed and resuspended in PBS. The analysis was performed with a Becton Dickinson FACSCanto II Flow cytometer.

### Oxidative stress susceptibility assay

Susceptibility to oxidative stress using 2 mM and 20 mM paraquat was tested on Columbia agar plate using the disc method as described [32].

### Antibiotic Sensitivity Testing

Wild-type, Δ*xerH* mutant and *xerH* complemented strains were inoculated on Columbia agar plates. A single E-test strip (AB Biodisk) was placed in the centre of each plate and the plates were incubated at 37°C for 6 days. The minimal inhibitory concentration (MIC) for ciprofloxacin was determined according to the manufacturer's instructions.

### Growth curves

Fresh cultures, passaged the day before, were resuspended in BHI. 5 ml of BHI containing 10% NCS and Dent (Oxoid) was inoculated with 100 µl of a stock inoculum standardized to OD_600_ = 2. Growth studies were performed without any prior adaptation of *H. pylori* strains to liquid media. Growth was measured every 4 to 12 h for up to 40 h. Each experiment was done in duplicate and repeated at least twice.

### Electron microscopy

*H. pylori* grown on Columbia agar were collected and washed in PBS (pH 7.4), prefixed in 2.5% glutaraldehyde in PBS buffer for 1 h, and then rinsed in PBS. After post-fixation in 1% osmium tetraoxide (in PBS), samples were dehydrated through ascending gradient of ethanol and then critical-point dried using carbon dioxide. Samples were sputter coated with palladium (4 nm) and examined using a SEM (Zeiss 1555 VP SEM) at 3 KV and a working distance of 6 mm.

### Phylogeny of the single Xer recombinases

Phylogenetic analysis of the Xer recombinase proteins was performed with MEGA version 4 [33]. Sequences were aligned with ClustalW, and the phylogeny was built using the Neighbor-Joining method [34].

### Experimental Infection of Mice

*Helicobacter* free C57BL/6J mice were purchased from the Animal Resource Centre (Perth, Western Australia). Studies were performed with approval from the UWA Animal Ethics Committee (approval no. 07/100/598). Each eight-week-old mouse was orogastrically inoculated with approximately 10^9^ CFUs of *H. pylori* harvested from an overnight agar plate culture into BHI broth. Colonisation of mice inoculated with X47 wild-type or its *ΔxerH* mutant was evaluated 2 weeks after challenge as described [29].

## Results

### The *difH* sequence undergoes site-specific recombination in *H. pylori*

An excision assay in *H. pylori* strain 26695 was designed to mimic chromosome dimer resolution (Figure 1A) and to test the ability of the recently identified *difH* site [14] to undergo site-specific recombination. This assay used a DNA segment with direct repeats of *difH* flanking *rpsL* and *cat* genes, which confer susceptibility to streptomycin and resistance to chloramphenicol, respectively. In our first tests this “*difH* repeat” cassette was inserted in the *H. pylori* chromosome between genes HP0203 and HP0204, which is about 525 kb from the normal *difH* site (Figure S1). A negative control strain contained an equivalent cassette, but with 40-bp of other DNA (‘*nondif*’) in place of *difH* at this same chromosomal location. In PCR tests, each of two bacterial clones containing the *difH* repeat cassette yielded two DNA fragments: 2.1 kb, expected of recombination between *difH* sites; and 3.6 kb, the full-length cassette (not recombinant at *difH* sites) (Figure 1B), whereas clones containing the *nondif* repeat cassette yielded only the 3.6 kb PCR product (Figure 1B). This outcome indicates that *difH* sites placed at a new chromosomal locus can undergo site-specific recombination.

*difH* recombination at the HP0203-HP0204 locus was also characterised by restoration of streptomycin resistance, which results from loss of the *rpsL* (susceptible) allele (*difH* recombination), or from rare gene conversion between the added *rpsL* gene in this cassette and the resistant allele at the normal chromosomal *rpsL* locus [22]. Under our conditions, no (<0.1%) streptomycin resistant colonies were obtained when the 40 bp yeast DNA (‘*nondif*’) sequence flanked the *rpsL-cat* cassette. In contrast, many streptomycin resistant colonies were obtained in strains in which *difH* flanked *rpsL-cat*. All of these streptomycin resistant colonies were chloramphenicol sensitive. PCR confirmed that streptomycin sensitive clones still contained the full-length *difH* repeat cassette and that *rpsL-cat* was absent from streptomycin resistant clones. As expected, DNA sequencing confirmed that a single *difH* “scar” sequence had been retained in these streptomycin resistant, chloramphenicol sensitive excisants, (Figure 1 and data not shown). The frequency of XerH recombination at *difH* sites at the *ureAB* locus (about 647 kb from *difH* site; Figure S1) was also evaluated using this *difH* repeat cassette. Cells were grown for 2 and 4 days in non-selective medium (no chloramphenicol), streaked out for single colony isolates, and 100–200 colonies were then tested for streptomycin/chloramphenicol resistance/susceptibility phenotypes. Figure 1C shows that the *difH* recombination frequency is significantly higher for the cassette placed at *ureAB* than at HP0203-HP0204 after 4 days culture. We conclude that the chromosomal position of paired *difH* sites affects the frequency of recombination between them.

### *difH* site recombination requires the single XerH recombinase

**Figure 2:**
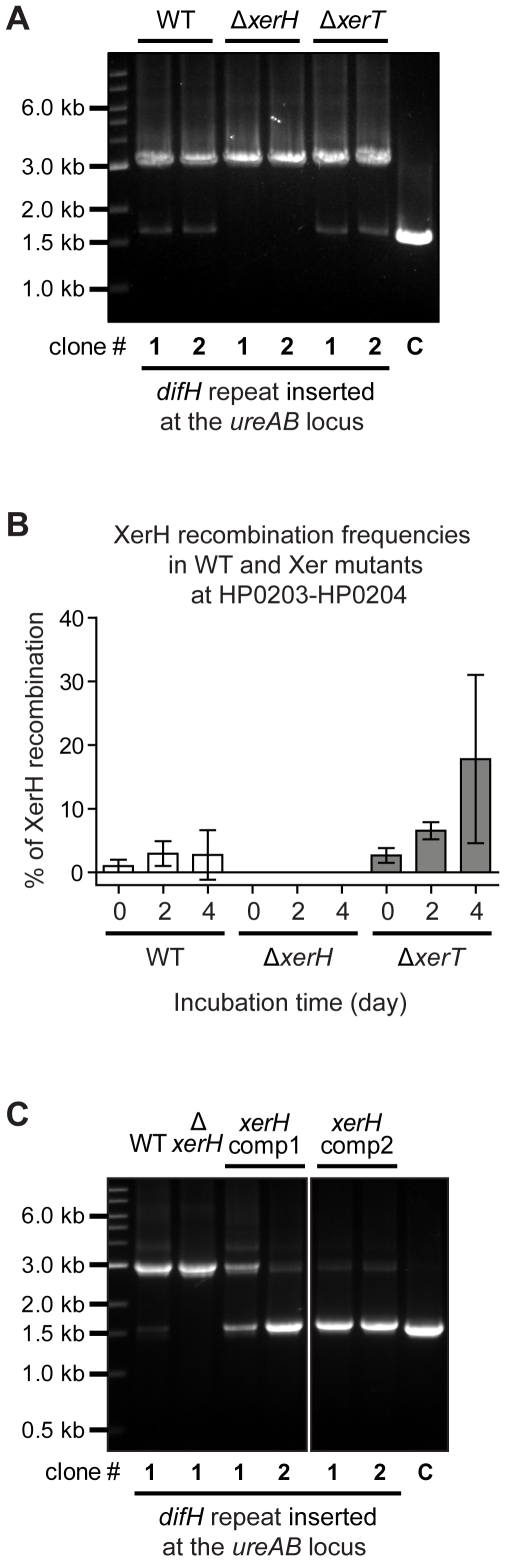
XerH is required for recombination at *difH* sites whereas XerT is not. PCR-based *difH* recombination was assayed in *H. pylori* 26695^Str^ (WT) and isogenic derivatives harbouring the *difH* repeat cassette after growth on non-selective media, essentially as in Figure 1. Two independent transformant clones of each strain were tested. The PCR product from control DNA (lane C), which did not have a *difH* repeat cassette, is slightly smaller than the product reflecting *difH* recombination because it does not contain the single copy of *difH* that remains after recombination (See Figure 1A). (A) Tests of *difH* recombination in WT and Δ*xerH* and Δ*xerT* derivatives, harbouring *difH* repeat cassette at the *ureAB* locus. (B) Tests of *difH* recombination in WT, *ΔxerH*, and *ΔxerT* derivatives, harbouring *difH* repeat cassette at the HP0203-HP0204 locus strains. (C) Tests of *difH* recombination in WT, Δ*xerH* and Δ*xerH* complemented with a highly expressed *xerH* gene using strains with the *difH* cassette at the *ureAB* locus.

*xerH* was the only xer recombinase gene identified computationally in every fully sequenced genome of the Campylobacterales order, which includes the genus *Helicobacter* [14]. However, many *H. pylori* strains also contain another *xer*-like recombinase gene named *xerT* (named for its association with the TnPZ transposon) [20], [21]. In frame deletions were made in both *xerH* and *xerT* to test each of them for possible roles in *difH* site-specific recombination. Using the *difH* repeat cassette at the *ureAB* locus and our PCR assay, we found that *xerH* deletion blocked *difH* recombination, whereas *xerT* deletion did not (Figure 2A). In addition, no excision was detected in the Δ*xerH* mutant by testing ∼200 colonies for the emergence of streptomycin resistant, chloramphenicol sensitive colony phenotypes after 2 and 4 days growth on non-selective medium, whereas the isogenic Δ*xerT* mutant strain has undergone as much recombination as the *xer*-wild-type strain, or possibly more, after 2 and 4 days incubation (Figure 2B).

Complementation of the *ΔxerH* allele with an intact *xerH* gene under a strong urease promoter (where it is probably over-expressed) restored *difH* site-specific recombination (Figure 2C). Indeed, the high level of the small PCR product in XerH complemented strains (*difH* recombination product; >80% of total) suggested that the amount of XerH protein per cell may be tightly controlled, and that increased XerH protein markedly increased the excision frequency, at least for *difH* at ectopic sites.

The plasmid used to introduce the *difH* repeat cassette into the *H. pylori* chromosome (pHInt_difH-RCAT-difH) did not undergo *difH* site recombination in *E. coli* (data not shown), indicating that *E. coli*'s own XerC and XerD recombinases do not process *difH*; this was as expected, given *difH* and *E. coli dif* sequence divergence (Table 1 and [14]). However, expression of XerH in *E. coli* promoted excision at *difH* sites in 10% of this plasmid population (estimate based on plasmid restriction enzyme digest profile; data not shown). Taken together, our results indicate that recombination between *difH* sites is mediated by just one Xer recombinase without other species-specific factors and that XerH may be limiting, at least for *difH* sites far removed from their normal location. They further suggested that XerT might inhibit XerH, since more *difH* recombination was seen in the *ΔxerT* strain than in its wild-type parent (Figure 2B), although further testing is needed to learn if the stimulation seen in a *ΔxerT* strain reaches statistical and thus biological significance.

**Table 1:** Consensus *difH* sequence obtained from complete *H. pylori* genomes.

*H. pylori* strains	*difH* sequences
35A	AAAATTCATTTAGTTATGAAAACTACACTTTTCAAACTTTTAAATCTAAC
51	AAAATTCATTTAGTTATGAAAACTGCACTTTTCAAACTTTTAAATCAAAC
52	ATTTTCTTGTTAGTTATGAAAACTACACTTTTCAAACTTTTAAATCTAAC
83	AAAATTCATTTAGTTATGAAAACTGCACTTTTCAAACTTTTAAATCAAAC
908	AAATCTATTTTAGTTATGAAAACTGCACTTTTCAAACTTTTAAATCAAAC
2017	AAATCTATTTTAGTTATGAAAACTGCACTTTTCAAACTTTTAAATCAAAC
2018	AAATCTATTTTAGTTATGAAAACTGCACTTTTCAAACTTTTAAATCAAAC
26695	TAAATTCATTTAGTTATGAAAACTGCACTTTTCAAACTTTTAAATCAAAC
B8	AAATCTATTTTAGTTATGAAAACTATACTTTTCAAACTTTTAAATCCAAC
B38	TAATCTATTTTAGTTATGAAAACTGCACTTTTCAAACTTTTAAGTCAAGC
Cuz20	AAAATTCATTTAGTTATGAAAACTGCACTTTTCAAACTTTTAAATCTAAC
F16	AAAATTCATTTAGTTATGAAAACTGCACTTTTCAAACTTTTAAATCAAAC
F30	AAAATTCATTTAGTTATGAAAACCGCACTTTTCAAACTTTTAAATCTAAC
F32	AAAATTCATTTAGTTATGAAAACTGCACTTTTCAAACTTTTAAATCTAAC
F57	AAAATTCATTTAGTTATGAAAACTGCACTTTTCAAATTTTTAAATCTAAC
G27	AAATCTCTTTTAGTTATGAAAACTGCACTTTTCAAACTTTTAACTCTAAC
Gambia94/24	AAATCTATTTTAGTTATGAAAACTGCACTTTTCAAACTTTTAAATCAAAC
HPAG1	AAATCTCTTTTAGTTATGAAAACTGCACTTTTCAAACTTTTAAGTCAAAC
India7	AAAATTCATTTAGTTATGAAAACTGCACTTTTCAAACTTTTAAATCAAAC
J99	AAATCTCTTTTAGTTATGAAAACTACACTTTTCAAACTTTTAAATCAAAC
Lithuania75	AAAATTCATTTAGTTATGAAAACTGCACTTTTCAAACTTTTAGATCAAAC
P12	AAAATTCATTTAGTTATGAAAACTGCAATTTTCAAACTTTTAAATCAAAC
PeCan4	AAAATTCATTTAGTTATGAAAACTGCACTTTTCAAACTTTTAAATCTAAC
Puno135	AAAATTCATTTAGTTATGAAAACTGCACTTTTCAAACTTTTAAATCTAAC
Puno120	AAAATTCATTTAGTTATGAAAACTGCACTTTTCAAACTTTTAAATCTAAC
Sat464	AAAATTCATTTAGTTATGAAAACTGCACTTTTCAAACTTTTAAATCTAAC
Shi470	AAAATTCATTTAGTTATGAAAACTACACTTTTCAAACTTTTAAATCTAAC
SJM180	AAATCTATTTTAGTTATGAAAACTGCACTTTTCAAACTTTTAAGTCAAGC
SNT49	AAAATTCATTTAGTTATGAAAACTGCACTTTTCAAACTTTTAAATCAAAC
SouthAfrica7	TAATTTATTTTAGTTATGAAAACTGCACTTTTCAAACTTTTAAATCAAAC
v225d	AAAATTCATTTAGTTATGAAAACTGCACTTTTCAAACTTTTAAATCTAAC
	---------***************--*-********-*****--**-*-*
*H. pylori difH* consensus(1)	WWWWYYHWK**TTAGTTATGAAAACTRY** **A**M**TTTTCAAA**Y**TTTTA**RV**TC**H**A**R**C**
	------------**----*-**------**-*---*--*-----------
*E. coli dif* consensus(2)	-----------GGTGCGCATAATGTATATTATGTTAAATC----------

(1) *difH* sequences were retrieved from *H. pylori* complete genome sequences downloaded from the National Center for Biotechnology Information (http://www.ncbi.nlm.nih.gov).

(2) Blakely GW, Davidson AO, Sherratt DJ (1997) Binding and cleavage of nicked substrates by site-specific recombinases XerC and XerD. J Mol Biol 265: 30–39.

### Point mutations in *difH* sequences that block XerH-mediated recombination

**Figure 3:**
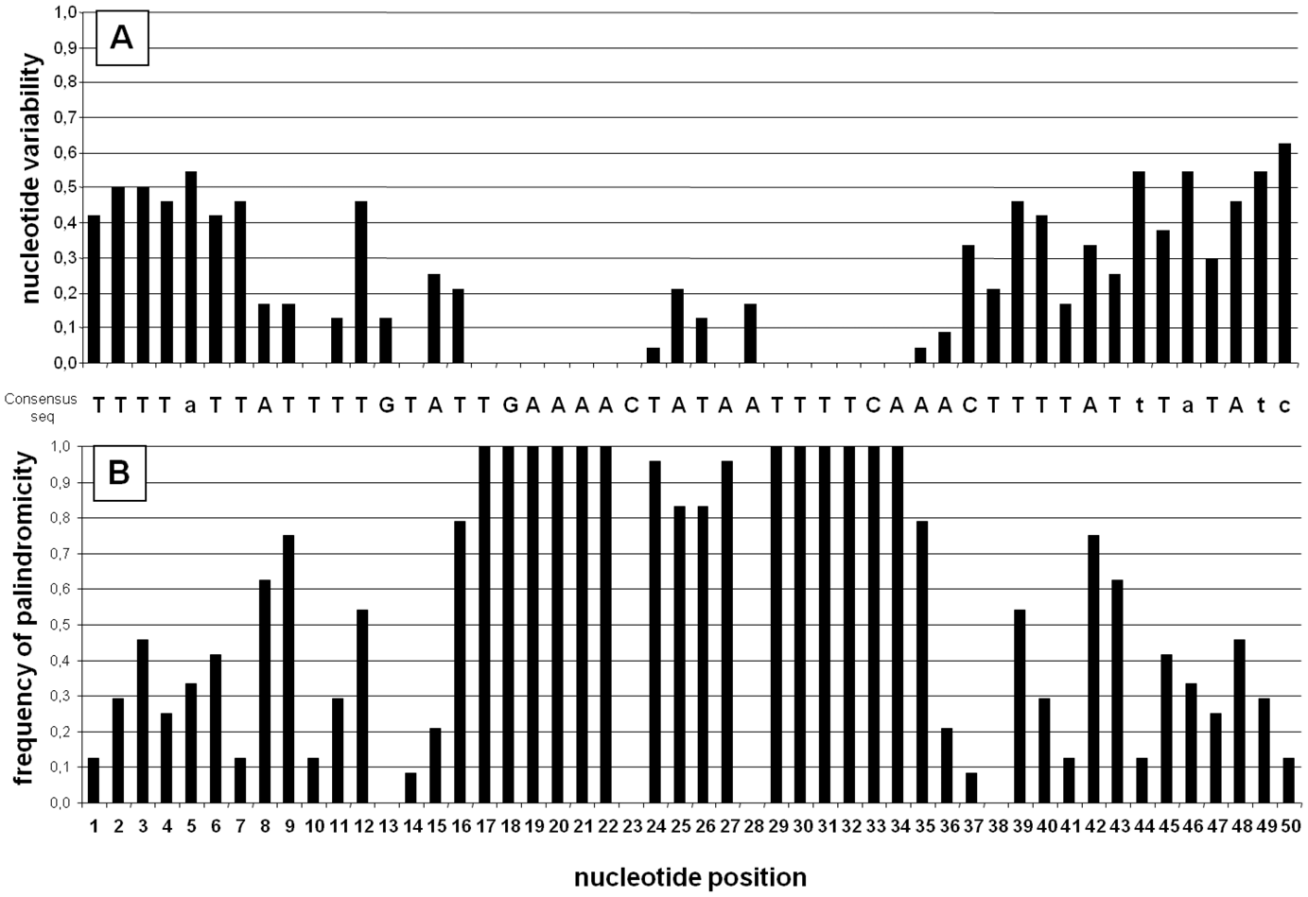
*difH* sequences. (A) *difH* consensus sequence and nucleotide variability for *difH* sequences from 24 epsilon- proteobacterial species (Table 2). If a given nucleotide is present in more than 50% of species it is written in upper case; if not, the most frequent nucleotide is in lower case. The nucleotide variability at each position was defined as 1–f, where f is the frequency of the most frequent nucleotide. (B) Palindromicity was analysed by comparing the 50-nt *difH* sequence with its inverted complementary counterpart in the 24 epsilon-proteobacterial species (Table 2). When a nucleotide was found both in *difH* and in the reverse complementary sequence, a value of 1 was given to the position. Next, the values for the 24 *difH* sequences for each position were added together to give the *n* value. The palindromicity frequency (*fpal*) was then estimated as: *fpal* = *n*/24, with 24 being the number of *difH* sequences analysed. A *fpal* value of 1 given to a nucleotide position means that the nucleotide is always part of a palindrome.

Before performing a mutational analysis of the *difH*, the nucleotide frequency at each position was calculated over 50 bp from 24 epsilon proteobacterial species to establish a consensus sequence (Table 2, Table S3). This revealed that *difH* consists of two highly conserved regions (position 17 to 23 and 29 to 34) separated by a variable region (position 24 to 26 and 28) and two other highly conserved positions, 10 and 14 (Figure 3A). In addition, positions 17 to 22 and 29 to 34 are always in a palindrome (inverted repeat), whereas positions 13, 23, 28, and 38 are not (Figure 3B). Thus, the *difH* sequence can be viewed as two matched domains flanking a unique 6 bp central region.

**Table 2:** Consensus *dif_H_* sequence obtained from 24 epsilon proteobacteria species.

	Species(1)	*difH* sequences(2)
Campylobacteraceae		
Arcobacter:	*Arcobacter butzleri* RM4018	AAATATTAATTAGTAT**TGAAAAC**TAT**A**A**TTTTCA**AATAAAATATAATAAA
	*Arcobacter nitrofigilis* DSM 7299	ATATATTAATTAGTAT**TGAAAAC**TAT**A**A**TTTTCA**AACTAAATATAGTTTT
Campylobacter:	*Campylobacter coli* RM2228 (uncompl.)	ATTAATTATTTTGTAT**TGAAAAC**TAT**A**A**TTTTCA**AACTTTTATATTTATA
	*Campylobacter concisus* 13826	TTTATATATTTTGTAT**TGAAAAC**TAT**A**A**TTTTCA**AATTGATATTTTAAAA
	*Campylobacter curvus* 525.92	TTTTTATATTTTGTAT**TGAAAAC**TAT**A**A**TTTTCA**AATTAATATTTATTTT
	*Campylobacter fetus subsp.* *fetus* 82-40	TTTTATTATTTTGTAT**TGAAAAC**TAT**A**A**TTTTCA**AACTATTATGAATTCT
	*Campylobacter gracilis* RM3268 (uncompl.)	TCTAGATATTCTATAT**TGAAAAC**TAT**A**A**TTTTCA**AGTAAAAATTCAATAC
	*Campylobacter hominis* ATCC BAA-381	TAAATTATTTTATTTT**TGAAAAC**TAT**A**A**TTTTCA**AACTTTTTTGTATTTT
	*Campylobacter jejuni* RM1221	ATTTATAATTTTGTAT**TGAAAAC**TGT**A**A**TTTTCA**AACTTTTTTATATACA
	*Campylobacter lari* RM2100	TGTATATATTTTGTAT**TGAAAAC**TAT**A**A**TTTTCA**AACTATTATATTTATC
	*Campylobacter rectus* RM3267 (uncompl.)	TTTTGCTATTTTGTAT**TGAAAAC**TGT**A**A**TTTTCA**AATAAATATCGATACC
	*Campylobacter showae* RM3277 (uncompl.)	TTTTGCTATTTTGTAT**TGAAAAC**TAT**A**A**TTTTCA**AATAAATATTTATATC
	*Campylobacter upsaliensis* RM3195 (uncompl.)	TTTTATAATTTTGTAT**TGAAAAC**TAT**A**A**TTTTCA**AACTTTTATTAAAACT
Sulfurospirillum:	*Sulfurospirillum deleyianum* DSM 6946	CAACTTCATTAATTAT**TGAAAAC**TAA**A**A**TTTTCA**AAATTACATAGTTATA
Helicobacteraceae		
Helicobacter:	*Helicobacter acinonychis* str. Sheeba	AAAAATAGTTTAGTTA**TGAAAAC**TGC**A**C**TTTTCA**AACTTTTAAATCAAAC
	*Helicobacter canadensis* MIT 98–5491 (uncompl.)	TTCTAATATTTTGTAT**TGAAAAC**TAT**A**A**TTTTCA**AACTTTTATTTTTAAC
	*Helicobacter cinaedi* CCUG 18818 (uncompl.)	TAACATAATTTAGTTA**TGAAAAC**TAT**A**C**TTTTCA**AACTTTTTTCCATTAT
	*Helicobacter hepaticus* ATCC 51449	GTGTTTGAATTAGTTA**TGAAAAC**TAT**A**C**TTTTCA**AACTTTTTTATCTCAA
	*Helicobacter mustelae* 12198	TAGTAAAATTAAGTTA**TGAAAAC**TGT**A**A**TTTTCA**CTAAAATAAATTTTTC
	*Helicobacter pullorum* MIT 98–5489 (uncompl.)	ACTCTATATTTTGTAT**TGAAAAC**TAT**A**A**TTTTCA**AACTTTTTTTGAAGGA
	*Helicobacter pylori* 26695	TAAATTCATTTAGTTA**TGAAAAC**TGC**A**C**TTTTCA**AACTTTTAAATCAAAC
	*Helicobacter winghamensis* ATCC BAA-430 (uncompl.)	TCTATCATTTTTGTAT**TGAAAAC**TAT**A**A**TTTTCA**AACTTTTTTGTTTCTT
Wolinella:	*Wolinella succinogenes* DSM 1740	GTATCTCATTTAGTAT**TGAAAAC**CAT**A**A**TTTTCA**AACTCATAATTGAATC
Nitratiruptor	*Nitratiruptor sp*. SB155-2	CCATATTTATTAGTAT**TGAAAAC**TAT**A**A**TTTTCA**AACTTTTATTTTTGTT
**CONSENSUS** (3)		TTTTaTTATTTTGTAT**TGAAAAC**TAT**A**A**TTTTCA**AACTTTTATtTaTAtc

(1) Uncompleted genome sequences were noticed as “uncompl.”.

(2) Nucleotides in bold characters are common to all 24 *dif_H_* sequences.

(3) If the nucleotide frequency represents more than 50%, the nucleotide is written in upper case letters; otherwise, the nucleotide is written in lower case letters.

**Figure 4:**
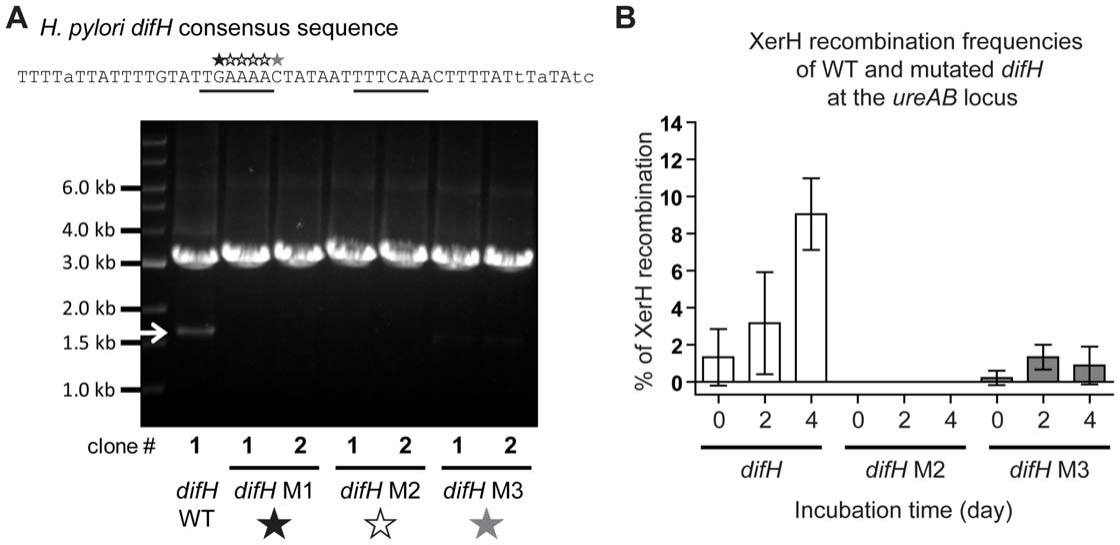
XerH-mediated recombination at mutant *difH* sequences. (A) The four A's in positions 19 to 22 of the *difH* sequence were changed to T's (empty stars), the G in position 18 was changed to C (black star) and the C in position 23 was changed to G (grey star). PCR tests were carried out as in Figure 1 on a clone harbouring WT *difH* repeats and two clones harbouring each mutant *difH* sequence (same sequence in each copy of *difH*) as indicated by the stars, *difH* M1, *difH* M2 and *difH* M3. No PCR product reflecting *difH* recombination was detected in *difH* M1, *difH* M2 mutants, whereas a weak band reflecting *difH* recombination was detected in the two clones with *difH* M3 sequences. Underlined nucleotides were found in a palindrome in all species studied. (B) *difH* recombination frequencies for WT *difH* and the *difH* M1, *difH* M2 and *difH* M3 mutant sequences carried out as in Figure 2B. Experiments were performed in triplicate; horizontal bars indicate means and standard deviations.

*difH* point mutations were made and tested for their ability to undergo *difH* recombination in *H. pylori* when at the *ureAB* locus. PCR tests showed that replacement of G by C in palindrome position 18 or of the four As by four Ts in palindrome positions 19 to 22 each abolished *difH* recombination in each of two independent clones (Figure 4A, lanes *difH* M1 and *difH* M2). *difH* recombination also seemed to be reduced by a C to G mutation at the non-palindromic but highly conserved position 23, although this mutation's effect seemed leaky (very weak excisant PCR product; Figure 4A, lane *difH* M3). In confirmation, tests for the appearance of streptomycin resistant clones showed that the *difH* M2 mutation abolished *difH* recombination, and that the *difH* M3 mutation caused a severe impairment of recombination (1±1% for *difH* M3 vs. 9±2% for *difH*–wild-type after 4 days incubation on non-selective medium; Figure 4B).

### FtsK is required for XerH recombination

**Figure 5:**
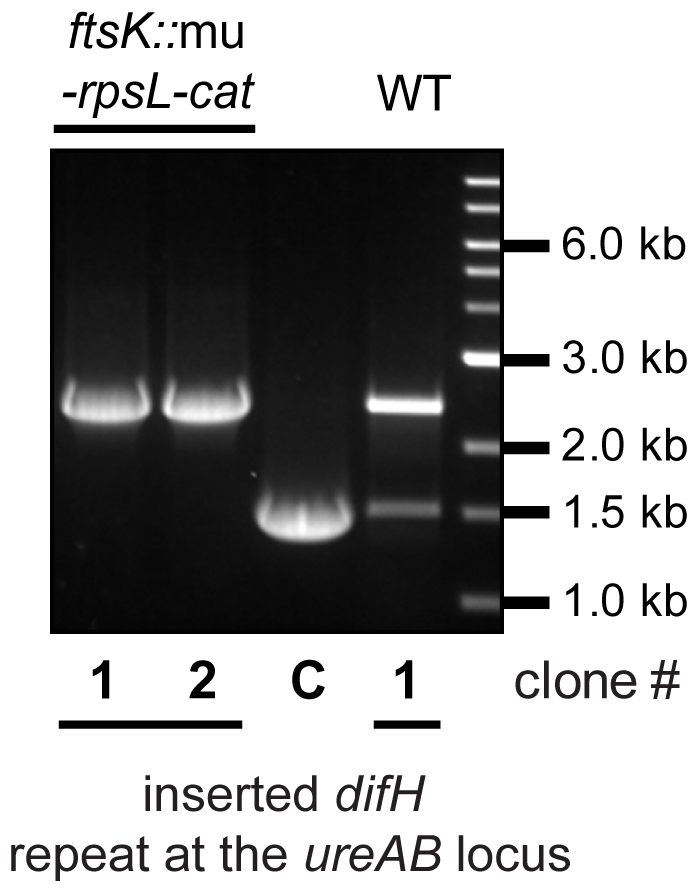
*H. pylori* FtsK is required for XerH-mediated recombination. Recombination at *difH* sites was scored as in Figure 2A in *H. pylori* 26695^Str^ (WT) and its derivative containing a C terminal deletion in *ftsK*, in each case with the *difH* repeat cassette at the *ureAB* locus.

The presence of an *ftsK* homologue (HP1090) in *H. pylori* suggested that XerH action might require the FtsK DNA translocase, much as does XerC/D action in *E. coli*. To test the role of *H. pylori*'s putative FtsK translocase protein, we deleted the 3′ terminal 1212 bp of the 2580 bp *ftsK* gene to generate a strain whose FtsK protein is truncated, and missing the γ regulatory domain that in the *E. coli* mediates FtsK interaction specifically with XerD, and not with the related XerC recombinase, for XerC/XerD mediated *dif* recombination [2]. Our mutant protein retains the N-terminal membrane anchor domain, which is essential for viability [35]. No *difH* recombination was detected by PCR in an *H. pylori* strain containing our mutant *ftsK* gene and the *difH* repeat cassette at the *ureAB* locus (Figure 5). We conclude that XerH mediated recombination between *difH* sites depends on FtsK in *H. pylori*, even for *difH* sites far from the normal *difH* locus (Figure S1), and despite *H. pylori*'s use of a single Xer-type recombinase.

In *E. coli* FtsK guides chromosomal translocation and presentation of *dif* to the Xer complex by a set of asymmetric KOPS sequences (5′-GGGNAGGG) whose distribution is skewed toward *dif* and the replication terminus and polarized (most on leading DNA strand during bidirectional chromosome replication). Although this octamer is also abundant in *H. pylori* genomes, we think it is unlikely to serve as KOPS for *H. pylori*'s FtsK protein because its genomic distribution is neither skewed near *difH* nor highly polarized. The only *H. pylori* octamer mimicking *E. coli*'s KOPS in its distribution is 5′-AGTAGGGG-3′ (Figure S1). This octamer was identified earlier by Hendrickson and Lawrence in their survey of many bacterial genomes [6] as a putative chromosome “architecture imparting sequence” for *H. pylori*. In accord with their view, we propose that it serves as *H. pylori* FtsK's guide for *difH* presentation, its KOPS sequence.

### Slight growth defect, UV and ciprofloxacin susceptibility, and resistance to oxidative stress of *ΔxerH* mutant

**Figure 6:**
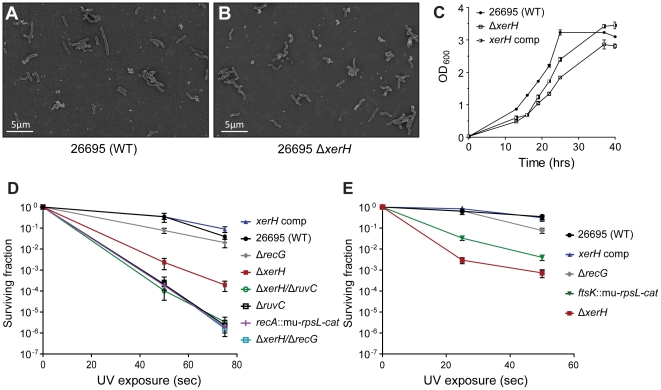
Phenotypes of *H. pylori* recombination mutants. (A) and (B) Electron micrographs. WT and Δ*xerH* mutant cells were fixed with glutaraldehyde and processed for scanning electron microscopy. Both WT and Δ*xerH* mutant cells displayed the characteristic curved rod morphology; in contrast to Δ*xer* mutant *E. coli*, none were filamentous. (C) Growth curves of *H. pylori* in liquid medium. Cells were grown in BHI liquid medium supplemented with 10% NCS in microaerobic conditions with agitation. The optical densities (OD_600 nm_) of WT, Δ*xerH* and complemented strains were measured in triplicate for up to 40 h. (D) UV sensitivity of WT and recombination mutant *H. pylori*. Cells were exposed to UV light as described in the methods and viable colony forming units (survival) was determined. Each test was repeated at least three times; standard deviation is indicated. (E) UV sensitivity of WT and *ftsK* mutant *H. pylori* determined as in part D.

Light and scanning electron microscopy indicated that loss of *xerH* did not cause filamentation in *H. pylori* equivalent to that caused by loss of *xerC* or *xerD* in *E. coli* (Figure 6A and 6B). The Δ*xerH* mutant grew less well than its wild-type parent did (Figure 6C), as did a derivative of this Δ*xerH* strain complemented by a highly expressed intact *xerH* gene (Figure 6C). These outcomes suggest that XerH levels are regulated – that either too much or too little can be deleterious.

Studies in *E. coli* showing that *xerC* inactivation exacerbated the moderate UV susceptibility that is caused by a *ruv*-deficiency [36] prompted us to test if *xerH* inactivation affects *H. pylori*'s UV susceptibility. Figure 6D shows that *xerH* inactivation caused UV sensitization, albeit less extreme than that caused by *recA* or *ruvC* inactivation, and that normal UV resistance was restored to a *ΔxerH* mutant by complementation with a functional *xerH* gene. Inactivation of *xerH* in a Δ*ruvC* mutant did not affect this strain's normally very high UV sensitivity, whereas enhanced UV sensitivity was observed in a *ΔrecG* mutant with increased recombination [37] compared to either mutant alone (Figure 6D). The Δ*recA ΔrecG* or Δ*ruvC ΔrecG* double and Δ*xerH ΔruvC ΔrecG* triple deletion strains had the same phenotype as Δ*recA* or Δ*ruvC* single deletion strains (data not shown). Two failed attempts to obtain a *ΔrecA* derivative of a Δ*xerH* strain prevented assessment of UV sensitivity in the absence of both *xerH* and RecA-mediated generalized recombination. Deletion of sequences encoding the FtsK gamma (probable XerH interaction) domain caused moderate UV sensitization, almost as much as that caused by *ΔxerH* itself (Figure 6E). Ciprofloxacin induces DNA double-strand breaks that are repaired by RecA- and RuvABC-mediated homologous recombination [38].The Δ*xerH* mutant was more sensitive to ciprofloxacin than wild-type (Table 3) despite *H. pylori*'s functional *recA* and *ruvABC* genes. This suggested a function other than DNA repair for XerH (e.g., dimeric and catenated chromosome resolution). The complemented *xerH* mutant (over-expressing XerH) was more resistant to ciprofloxacin (Table 3), again indicating a function other than DNA repair. Finally, the oxidative stress resistances of Δ*xerH* and Δ*ftsK* mutant *H. pylori* were similar to that of wild- type, as were Δ*recA*, Δ*ruvC* and Δ*recG* mutant strains (Figure S2). This outcome indicates that XerH recombination is not needed for base pair excision repair in *H. pylori*. Since the generalized recombination that UV, *recG* deletion and ciprofloxacin promote should result in formation of dimeric and catenated chromosomes, we suggest that failure to resolve such topological structures underlies the Δ*xerH* and *ΔftsK* mutant phenotypes. We propose that Xer recombination resolve chromosome dimers in *H. pylori* and speculate a role of XerH/*difH* in chromosome decatenation.

**Table 3:** Ciprofloxacin susceptibility of *H. pylori* strains.

Strains	MIC (µg/ml)^1^
	Median^2^
WT	0.125
Δ*xerH*	0.079
*xerH* complemented	0.250

(1) Minimum inhibitory concentration.

(2) 95% confidence interval based on the Wilcoxon signed rank test of eight independent experiments.

### Impaired chromosome segregation in *ΔxerH* mutant

**Figure 7:**
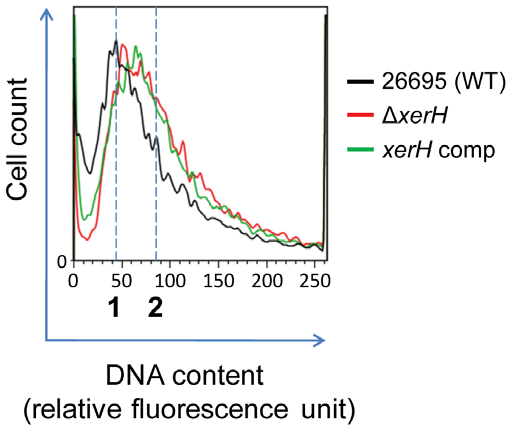
Impaired chromosome segregation in Δ*xerH* mutant. The X and Y axes indicate the relative Hoechst fluorescence units and number of *H. pylori* cells, respectively. Dotted vertical lines indicate genome equivalents. The main fluorescent signal of wild-type *H. pylori* was considered as one genome equivalent, as described [39].

As noted above, an inability to resolve chromosome dimers and catenated chromosomes should block chromosome segregation at cell division. The lack in *H. pylori* of homologues of *parC* and *parE*, which in *E. coli* encode the two subunits topoisomerase IV, suggested that XerH-mediated recombination might also be used for chromosome decatenation. To test this idea, the DNA contents of wild-type, Δ*xerH* mutant and complemented Δ*xerH* mutant strains were analysed by flow cytometry after staining of DNA with Hoechst dye, much as in other studies of the *hobA* chromosome replication initiation gene [39]. This showed that Δ*xerH* mutant cells contained more DNA on average than their wild-type parents did (Figure 7). In order to specifically test that XerH could perform decatenation, uncomplicated by chromosome dimers, which arise by generalized (RecA-mediated) recombination, we would have needed a *ΔrecA* Δ*xerH* double mutant. However, as noted above, we were unable to construct this double deletion strain.

We also note that the DNA contents of XerH complemented cells, which have increased XerH activity, was higher than those of isogenic wild-type cells (Figure 7). This suggests that excess XerH protein stimulates initiation of chromosome replication, or conceivably, that it interferes with chromosome segregation (reminiscent of that seen when XerH protein is absent). Taken together, these results suggest that Δ*xerH* mutants do not undergo efficient chromosome segregation, that they accumulate subpopulations, one with multiple (dimeric and perhaps entangled) chromosomes, and one without chromosomal DNA. A chromosome segregation defect is in line with a failure to resolve dimeric and catenated chromosomes, structures that Xer recombination can resolve in *E. coli* [7].

### XerH is needed for gastric colonisation

**Figure 8:**
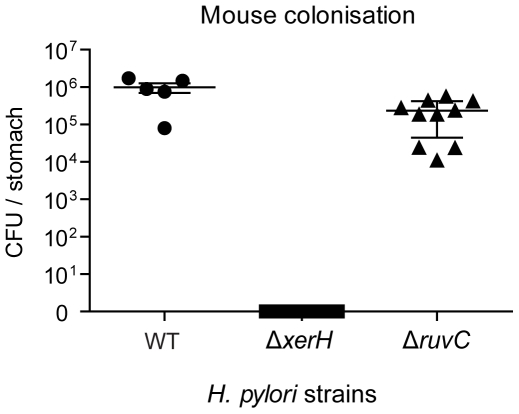
XerH is needed for gastric niche colonization. *H. pylori* X47 wild-type (WT) and isogenic Δ*xerH* and Δ*ruvC* mutants were used to inoculate five to ten eight-week old C57BL/6J mice. Mice were sacrificed and colonisation levels in stomachs were measured as described in the methods. Data is presented as a scatter plot with each point representing the CFU count of one mouse stomach, and the solid line the geometric mean ± standard deviation for each group (WT, Δ*xerH*, and Δ*ruvC*).

The *ΔxerH* mutant's apparent defect in chromosome segregation and lack of severe growth phenotype *in vitro* prompted us to test if *xerH* is needed by *H. pylori* in its gastric mucosal environment. The *ΔxerH* allele, and for comparison, a *ΔruvC* allele, were transformed into strain X47, which colonizes mice robustly. C57BL/6J mice were inoculated orogastrically with mixtures of these mutant strains and their isogenic X47 wild-type parent; the mice were sacrificed two weeks later and gastric mucosal levels of *H. pylori* were assayed by bacterial culture. Compared to the robust stomach colonization observed for wild-type *H. pylori* (WT), the Δ*xerH* mutant did not colonize mice at all (Figure 8). Interestingly, the Δ*ruvC* mutant, which exhibits a more severe DNA repair defect, did colonise mice, although with a 5-fold lower bacterial load than wild-type (Figure 8). The inability of the Δ*xerH* mutant to survive in the gastric niche contrasts with Δ*ruvC* mutant colonization, and further supports the idea that XerH is not involved in DNA repair, but rather in chromosome maintenance such as chromosome dimer resolution and possibly in chromosome unlinking. This, in turn, suggests that the slow growing *H. pylori* depends on a unique chromosome replication and maintenance machinery to thrive in its special gastric niche.

## Discussion

**Figure 9:**
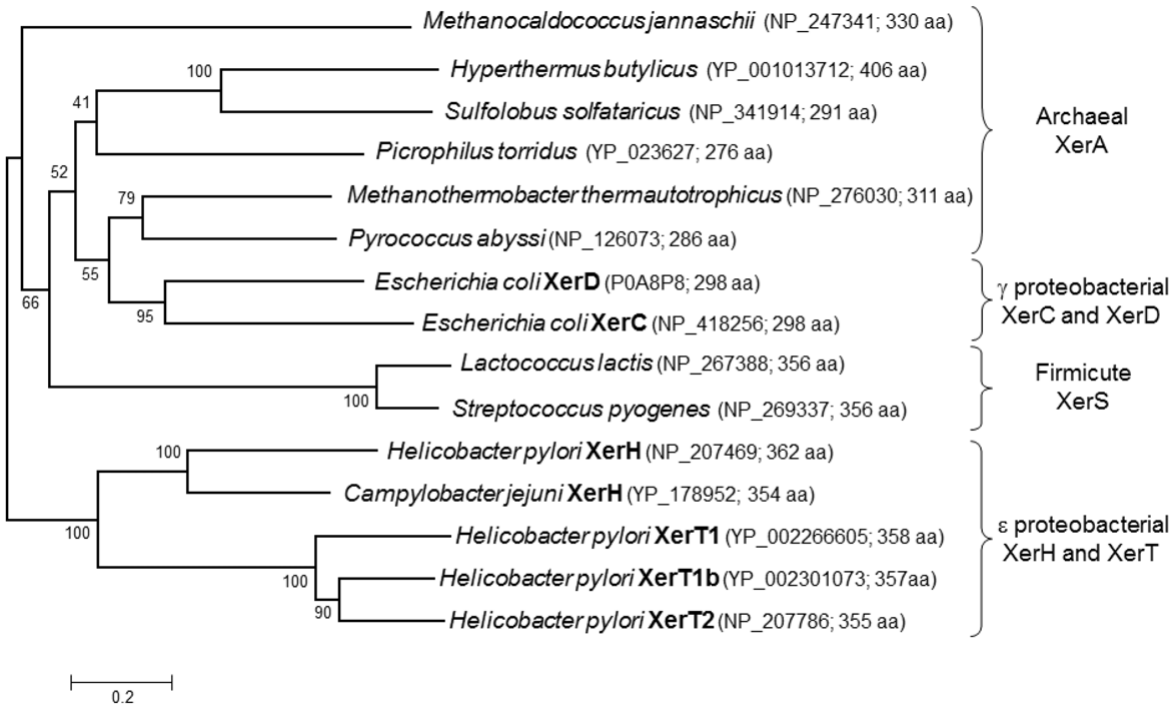
Phylogeny of archaeal and bacterial single-Xer recombinases. Species are representative of their respective taxonomic groups. XerC and XerD from *E. coli* and XerT from *H. pylori* were included in the study as reference. Amino acid sequence alignments were performed using Clustal W. The phylogenetic analyses, using the Neighbour-Joining method [34] were conducted in MEGA4 [33]. The percentage of replicate trees in which the associated taxa clustered together in the bootstrap test (1000 replicates) is shown next to the branches. The bootstrap consensus tree is taken to represent the evolutionary history of the taxa analysed. For each species, the accession number is indicated as well as the number of amino acid residues composing the recombinase.

The present study confirmed our computational prediction [14] that *H. pylori* uses just one dedicated tyrosine recombinase, XerH, for site-specific recombination at a cognate chromosomal *dif* site (*difH*) – not a pair of distinct proteins akin to XerC and XerD tyrosine recombinases of *E. coli* and most other eubacterial species. For our experiments, we constructed a cassette with direct repeats of *difH* sites flanking counterselectable and selectable genes (*rpsL* and *cat*, respectively) and placed this “*difH* repeat” cassette at arbitrarily chosen *H. pylori* chromosomal locations. *difH* recombination was detected by loss of the *rpsL-cat* segment, as scored by bacterial phenotype or PCR. Deletion of *xerH* blocked recombination between *difH* sites in the *H. pylori* chromosome; and conversely, *xerH* expression promoted recombination between them in a plasmid in *E. coli*. The related XerT recombinase, present in many but not all *H. pylori* strains [14], was not needed for *difH* recombination. This fits with XerT's usually being associated with a widespread conjugative transposon, but not a fixed component of every *H. pylori* genome [20], [21]. Single Xer proteins are also used for recombination at cognate *dif* sites in Lactococci [15], Streptococci [15] and related Gram positive genera [16], and in Archaea [17], [18], but they are distinct phylogenetically from *H. pylori*'s XerH and XerT (Figure 9).

Our Δ*xerH* mutant *H. pylori* exhibited a general DNA segregation defect. No typical filamentation was observed during normal growth (Figure 6), as in *E. coli xerC* or *xerD* or *dif* site mutant strains [40], or under UV stress (data not shown). This discrepancy may be explained by *H. pylori*'s lack of an SOS response and *E. coli*-type cell division inhibitor (SulA), which is induced by the DNA breakage [4] that occurs when cell division proceeds without chromosome dimer resolution. Alternatively, the lack of filamentation in *H. pylori ΔxerH* mutant might be ascribed to *H. pylori*'s much longer doubling time (3–4 hours) and small genome size (one-third *E. coli*'s). Although *E. coli* chromosome replication takes some 40 min, rapidly growing *E. coli* (20 min generation time) can undergo multifork replication. In consequence, rounds of replication can initiate in one cell cycle, finish in the next cell cycle, and still allow segregation in which each daughter cell receives at least one complete genome. There should be no such need for multifork replication in *H. pylori*, with its small genome size, and leisurely growth rate. We propose that these features underlie the lack of filamentation in Δ*xerH* mutant *H. pylori*.

Deletion of *xerH* in *H. pylori* caused: (i) a slight growth defect in liquid culture (Figure 6C), as is typical of *Δxer* mutants of *E. coli* [40] (ii) a markedly increased sensitivity to DNA breakage inducing and homologous recombination stimulating UV irradiation (Figure 6, D and E), and ciprofloxacin (Table 3), (iii) an increased UV sensitivity of a Δ*recG* mutant [37]; (iv) increased cellular DNA content (Figure 7), which we interpret as a defect in chromosome segregation; and (v) an inability to colonize mice (Figure 8). Overexpression of *xerH* in our complementation experiments also increased the level per cell. This unexpected finding suggests a role of XerH in regulation of DNA replication/segregation and merits further study. We also found that an intact FtsK DNA translocase protein was needed for *difH* recombination (Figure 5), presumably for effective *difH* site presentation to *H. pylori*'s XerH recombination complex at the end of each DNA replication cycle, and much as expected based on *E. coli* results [2], [16], [17], [41], [42], [43], [44]. Xer recombination likely depends on and is regulated by cognate FtsK proteins in all eubacterial species, although, curiously not in Archaea, since they lack obvious *ftsK* genes [2], [16], [17], . Deletion of *H. pylori* FtsK's cytoplasmic domain, including its putative γ segment, which probably interacts with XerH, led to a loss of *difH* site recombination. This implies FtsK-XerH interaction for coordinated control of chromosome dimer resolution and segregation to daughters at cell division. Our finding of XerH-dependent *difH* recombination in *E. coli* raises the possibility of low level FtsK-independent recombination in *H. pylori* as in Vibrios [43] or lack of FtsK-Xer interaction specificity as observed in Streptococci [41].

The importance of the wild-type *difH* sequence was confirmed by finding that each of our several *difH* sites mutations interfered with *difH* recombination (Figure 4). *difH*'s short inverted repeats, which flank a small central unique sequence spacer, have a slight asymmetry (e.g., left arm positions 10 and 14) that is well conserved among epsilon proteobacteria (Figure 3A). We speculate that this asymmetry could be used to determine the time and place of the FtsK-XerH complex's DNA cleavages: first on one *difH* strand, and then the other – reminiscent of the sequential cleavages by the phylogenetically distinct single XerS recombinase [41], and formally equivalent to the different roles and timing of action of *E. coli*'s XerC and XerD proteins in its heterodimeric recombinase [45]. Our computational analysis further identified the sequence 5′-AGTAGGGG, whose polarized clustering near *difH* make it a prime candidate for *H. pylori*'s KOPS; this octamer had also been noted earlier, and was formally proposed as a chromosome “architecture imparting sequence”, but without suggesting a molecular terms [6]. *H. pylori*'s putative KOPS diverges markedly from *E. coli*'s KOPS octamer (Figure S1), a feature that should encourage comparison of FtsK-KOPS binding and associated interactions in these two species.

The reason for our inability to delete *recA* in a *ΔxerH* mutant strain is unclear, but might suggest a second important role for *difH* recombination. For example, many thousands of topological links created by unwinding and replication of double stranded circular DNAs must all be removed for proper chromosome segregation at cell division. The great majority of such links are removed in *E. coli* by topoisomerase (Topo) IV, which interacts with and is stimulated by FtsK. However, *H. pylori* seems to lack this essential enzyme: it has no close homologues of the Topo IV encoding *parC* and *parE* genes [19], and thus must use some other enzyme system. The possibility that XerH/*difH* recombination could allow *H. pylori* to avoid a chromosome decatenation dilemma is suggested by findings of *E. coli* XerC/D and *dif*-dependent (although inefficient) DNA decatenation *in vitro*; and by the *in vivo* XerC- and XerD-dependent suppression of a temperature sensitive (conditional lethal) Topo IV mutation when mutant soluble form of FtsK is overproduced [7]. We think that the case for XerH-mediated decatenation in *H. pylori* would be strengthened if it were shown that this suppression reflects fulfilment of Topo IV's functions by a more effective XerC/XerD/FtsK complex, not just FtsK-mediated stabilization of an impaired (temperature sensitive) Topo IV [11]. It is interesting in this context, that obvious *parC* and *parE* (Topo IV) homologues are found in Lactococci and Streptococci, which also use just a single Xer recombinase, these Gram positive species do not challenge our conventional understanding of how chromosomes are decatenated [19] in the way that *H. pylori* does. As a third case, Archaea, use just a single Xer recombinase (XerA) but lack obvious homologues of *parC* and *parE* and *ftsK* [17]. Assuming that they will have developed yet another solution to the decatenation problem, valuable insights should emerge from detailed comparisons of daughter chromosome separation and chromosome integrity maintenance in diverse microbial species. One possible solution emerges from finding of higher decatenase activity in the DNA gyrases of *M. tuberculosis* and *M. smegmatis* than of *E. coli* [46], [47].

Although we can speculate that *H. pylori* chromosome decatenation is mediated by iterated round of XerH action on *difH*, we can also imagine DNA gyrase-mediated decatenation in *H. pylori*. This would be in accord with our *ΔxerH* strain's increased susceptibility to the gyrase inhibitor ciprofloxacin (Table 3), and *E. coli* DNA gyrase's low efficiency decatenation of linked circular DNAs *in vitro* (superimposed on its very efficient DNA negative supercoiling). As a final alternative, we can also imagine *H. pylori*'s topoisomerase III (HP0116) mediating sufficient decatenation, by extrapolation from Topo III's activity in *E. col*i [48].

The analyses presented here suggest many valuable experiments for future studies, bringing into focus the need to learn how catenanes are processed in the many other slow growing human pathogens that, like *H. pylori*, lack topoisomerase IV. Particularly informative should be further molecular genetic and enzymologic analyses of *H. pylori*'s XerH, DNA gyrase and TopoIII, in the context of this pathogen's small genome size and leisurely growth rate. The lessons learned should be applicable to the understanding, diagnosis and therapy for diverse pathogens and conditions: *H. pylori* itself, and peptic ulcer disease and gastric cancer; the closely related Campylobacters and associated diarrheal diseases; and equally, unrelated pathogens such as *Mycobacterium tuberculosis*, which chronically infects many millions of people worldwide, also without obvious genes for Topoisomerase IV.

## Supporting Information

Figure S1 **Positions of putative KOPS sequences and other features in the** ***H. pylori*** **26695 genome sequence.** The *H. pylori* 26695 genome sequence was scanned for the octameric AGTAGGGG sequences that had been implicated computationally as likely to affect chromosome architecture [6] (A), and for the GGGNAGGG octamer that constitutes the KOPS sequence of *E. coli* [49] (B). The circular *H. pylori* genome is presented here as a linear structure, with ends corresponding to its origin of bidirectional replication. The AGTAGGGG and GGGNAGGG octamers are represented by red and blue plain diamonds, respectively. Also indicated are the locations of *xerH* and *difH*, and the HP0203-HP0204 and *ureAB* loci at which we had placed *difH* repeat cassette.
(TIFF)

Figure S2 **Sensitivity of** ***H. pylori*** **mutants to oxidative stress.** Sensitivity to oxidative stress was evaluated in a disk assay using 2 mM or 20 mM of paraquat on blood agar plates that had previously been streaked for confluent growth with either mutant or wild-type cells as indicated. Following a 3–4 day incubation period, the clear zones surrounding the disks were measured. Experiments were repeated three times and standard deviation is indicated.
(TIF)

Table S1 **Plasmids and bacterial strains used in this study.**
(PDF)

Table S2 **Oligonucleotide primers used in this study.**
(PDF)

Table S3 **Nucleotide frequency (%) and consensus** ***difH*** **sequences from 24 epsilon-proteobacterial species chromosomes.**
(XLS)
