# Correction: Xer Recombinase and Genome Integrity in *Helicobacter pylori*, a Pathogen without Topoisomerase IV

**DOI:** 10.1371/annotation/f5629404-e0e6-4cc1-9d76-ab16238a48c0

**Published:** 2012-07-10

**Authors:** Aleksandra W. Debowski, Christophe Carnoy, Phebe Verbrugghe, Hans-Olof Nilsson, Jonathan C. Gauntlett, Alma Fulurija, Tania Camilleri, Douglas E. Berg, Barry J. Marshall, Mohammed Benghezal

There was an error in affiliation 1 for authors Aleksandra W. Debowski, Phebe Verbrugghe, Hans-Olof Nilsson, Jonathan C. Gauntlett, Alma Fulurija, Tania Camilleri, Barry J. Marshall, and Mohammed Benghezal. Affiliation 1 should be: Ondek Pty Ltd and Helicobacter pylori Research Laboratory, School of Pathology & Laboratory Medicine, M504, Marshall Centre for Infectious Disease Research and Training, University of Western Australia, Nedlands, Western Australia 

