# Supplementary figures and images for: Xer Recombinase and Genome Integrity in Helicobacter pylori, a Pathogen without Topoisomerase IV
Source: PLoS One. 2012 Apr 12;7(4):e33310. doi: 10.1371/journal.pone.0033310 (PMC3325230; doi:10.1371/journal.pone.0033310)

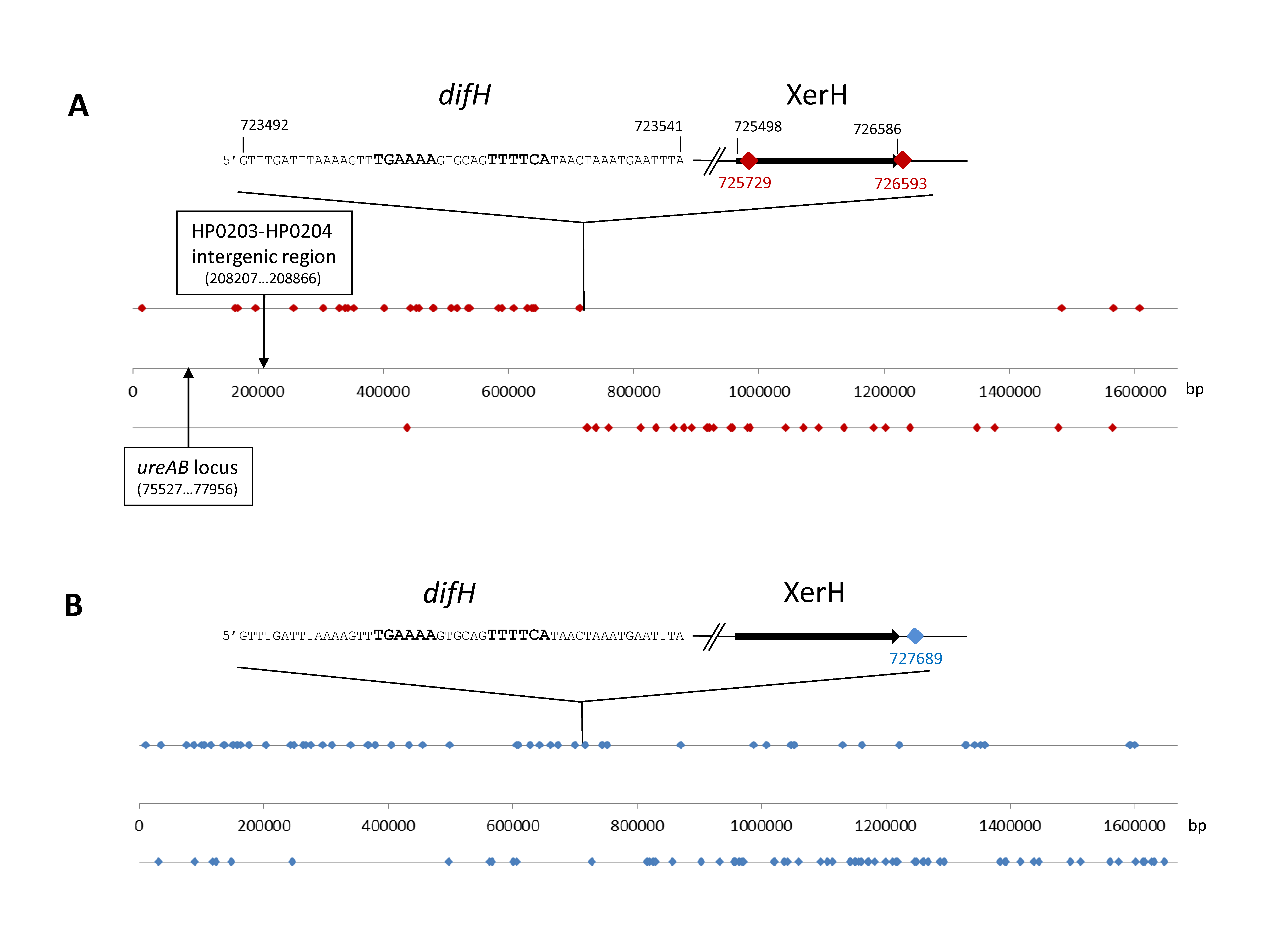

Supplement: Figure S1 — Positions of putative KOPS sequences and other features in the H. pylori 26695 genome sequence. The H. pylori 26695 genome sequence was scanned for the octameric AGTAGGGG sequences that had been implicated computationally as likely to affect chromosome architecture [6] (A), and for the GGGNAGGG octamer that constitutes the KOPS sequence of E. coli [49] (B). The circular H. pylori genome is presented here as a linear structure, with ends corresponding to its origin of bidirectional replication. The AGTAGGGG and GGGNAGGG octamers are represented by red and blue plain diamonds, respectively. Also indicated are the locations of xerH and difH, and the HP0203-HP0204 and ureAB loci at which we had placed difH repeat cassette. (TIFF) [file pone.0033310.s001.tiff]

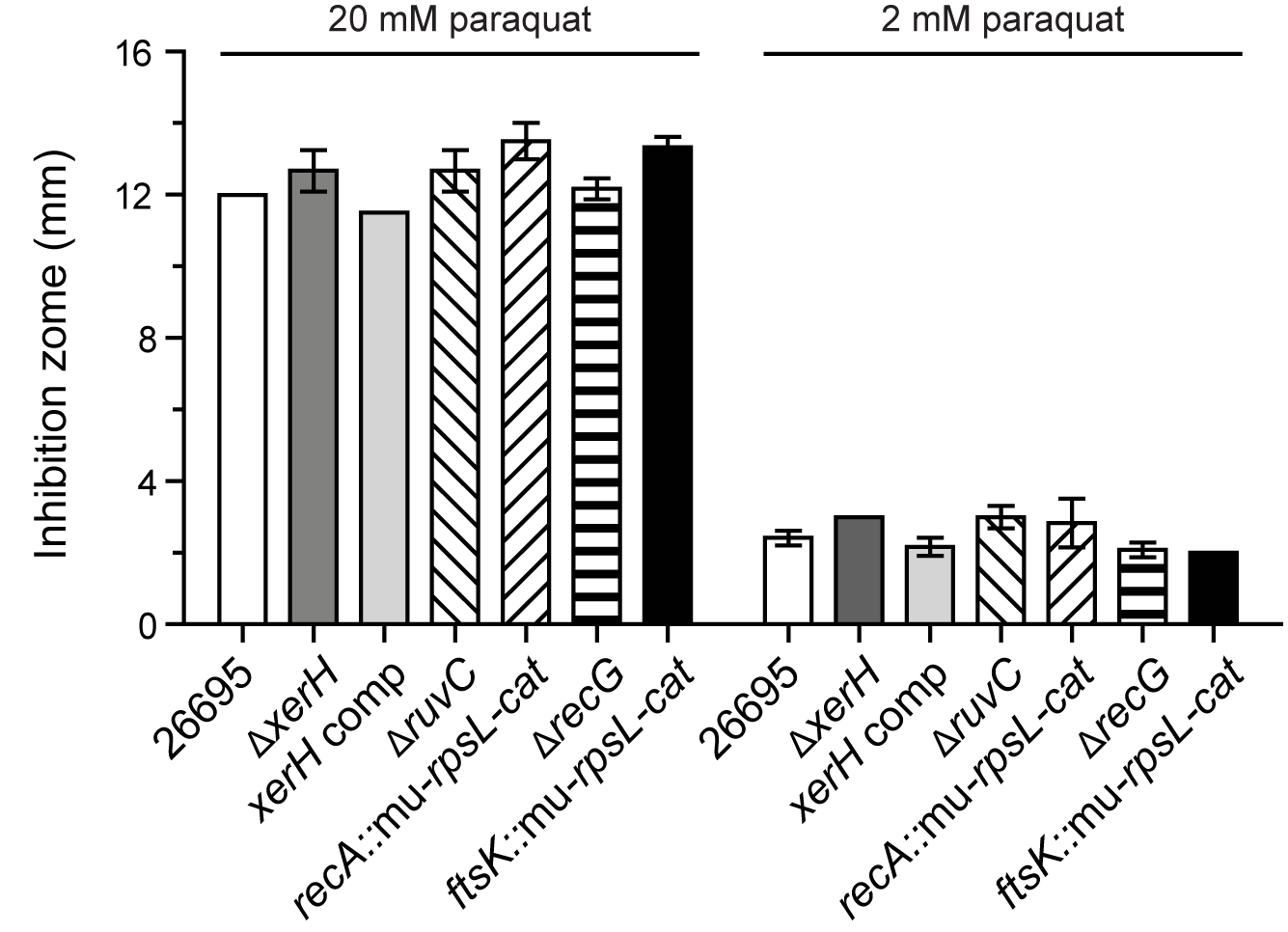

Supplement: Figure S2 — Sensitivity of H. pylori mutants to oxidative stress. Sensitivity to oxidative stress was evaluated in a disk assay using 2 mM or 20 mM of paraquat on blood agar plates that had previously been streaked for confluent growth with either mutant or wild-type cells as indicated. Following a 3–4 day incubation period, the clear zones surrounding the disks were measured. Experiments were repeated three times and standard deviation is indicated. (TIF) [file pone.0033310.s002.tif]
