# Supplementary material for: Xer Recombinase and Genome Integrity in Helicobacter pylori, a Pathogen without Topoisomerase IV
Source: PLoS One. 2012 Apr 12;7(4):e33310. doi: 10.1371/journal.pone.0033310 (PMC3325230; doi:10.1371/journal.pone.0033310)
Supplement: Table S1 — Plasmids and bacterial strains used in this study. (PDF) [file pone.0033310.s003.pdf]

Table S1. Plasmids and bacterial strains used in this study

Strain or plasmid	Description	Source or reference
Plasmid:		
pHSG576	Low copy plasmid	(Takeshita et al., 1987)
pHInt_difH-RCAT-difH	pHSG57 plasmid containing *difH-rpsL-cat-difH* cassette inserted between genes HP0203 and HP0204	This work
pHInt_nondif-RCAT-nondif	pHSG57 plasmid containing *nondif-rpsL-cat-nondif* cassette inserted between genes HP0203 and HP0204	This work
pDifWT	Wild-type *difH* sequence	This work
pDifM1	Mutant *difH* sequence 1 (GAAAAC -> CAAAAC)	This work
pDifM2	Mutant *difH* sequence 2 (GAAAAC -> GTTTTC)	This work
pDifM3	Mutant *difH* sequence 3 (GAAAAC -> GAAAAT)	This work
pDifWT-RC	*rpsL-cat* cassette flanked by *difH* WT	This work
pDifM1-RC	*rpsL-cat* cassette flanked by *difH* M1	This work
pDifM2-RC	*rpsL-cat* cassette flanked by *difH* M2	This work
pDifM3-RC	*rpsL-cat* cassette flanked by *difH* M3	This work
pUreAB	pBluescript plasmid containing 2kb of *ureAB* locus, with a 12 bp multiple cloning site inserted between the *ureA* and *ureB* ORFs	(Benghezal et al., 2009)
pUreAB_DifWT-RC	pUreAB with *difH*WT-*rpsL-cat-difH*WT cassette inserted between *ureA* and *ureB* ORFs	This work
pUreAB_DifM1-RC	pUreAB with *difH*M1-*rpsL-cat-difH*M1 cassette inserted between *ureA* and *ureB* ORFs	This work
pUreAB_DifM2-RC	pUreAB with *difH*M2-*rpsL-cat-difH*M2 cassette inserted between *ureA* and *ureB* ORFs	This work
pUreAB_DifM3-RC	pUreAB with *difH*M3-*rpsL-cat-difH*M3 cassette inserted between *ureA* and *ureB* ORFs	This work
pTrpA-up	pBluescript plasmid containing the *trpA* (HP1277) locus. Inserted into the centre of the *trpA* ORF is the core *ureA* promoter followed by 36 bp multiple cloning site.	This work
pTrpA-RC	pBluescript plasmid containing the *trpA* (HP1277) locus with *rpsL-cat* cassette	This work

	insertion	
pTrpA-upXerH	Variant of pTrpA-up, HP1277 with insertion of *xerH* fused to the core *ureA* promotor	This work
pHRecG	pHSG57 plasmid containing 1 kb of DNA sequences that flank the HP1523 ORF	This work
pHRecG-Km	Variant of pHRecG, containing HP1523 locus with HP1523 replaced by *aphA* cassette	This work
pRecA	HP0153 in pGEMT-Easy	This work
pRecA-RC	Variant of pRecA, *rpsL-cat* cassette	This work
pFtsK	HP1090 in pGEMT-Easy	This work
pFtsk-RC	Variant of pFtsK, *rpsL-cat* cassette inserted into HP1090 gene by Mu transposase	This work
H. pylori strain:		
26695	wild-type strain	(Akopyants et al., 1995)
26695^{Str}	Streptomycin resistant	(Dailidiene et al., 2006)
26695^{Str} *HP0203-4*::*difH-RCAT*	*difH-rpsL-cat-difH* cassette insertion between genes HP0203 and HP0204	This work
26695^{Str} *HP0203-4*::*nondif-RCAT*	*nondif-rpsL-cat-nondif* cassette insertion between genes HP0203 and HP0204	This work
26695^{Str} *xerH*::*rpsL-CAT*	HP0675 with *rpsL-cat* cassette insertion	This work
26695^{Str} *xerT*::*rpsL-CAT*	HP0995 with *rpsL-cat* cassette insertion	This work
26695^{Str} Δ*xerH*	HP0675 deletion	This work
26695^{Str} Δ*xerT*	HP0995 deletion	This work
26695^{Str} Δ*xerH*; *HP0203-4*:: *difH-RCAT*	HP0675 deletion and *difH-rpsL-cat-difH* cassette insertion between genes HP0203- HP0204	This work
26695^{Str} Δ*xerT*; *HP0203-4*:: *difH-RCAT*	HP0995 deletion and *difH-rpsL-cat-difH* cassette insertion between genes HP0203- HP0204	This work
26695^{Str} *ureAB*::*difH*WT-RC	*difH*WT-*rpsL-cat-difH*WT cassette insertion between *ureA* and *ureB*	This work
26695^{Str} *ureAB*::*difH*M1-RC	*difH*M1-*rpsL-cat-difH*M1 cassette insertion between *ureA* and *ureB*	This work
26695^{Str} *ureAB*::*difH*M2-RC	*difH*M2-*rpsL-cat-difH*M2cassette insertion between *ureA* and *ureB*	This work
26695^{Str} *ureAB*::*difH*M3-RC	*difH*M3-*rpsL-cat-difH*M3 cassette insertion between *ureA* and *ureB*	This work

26695^{Str} Δ*xerH*; *ureAB*::*difH*WT-RC	HP0675 deletion and *difH*WT-*rpsL*-*cat*-*difH*WT cassette insertion between *ureA* and *ureB*	This work
26695^{Str} Δ*xerT*; *ureAB*::*difH*WT-RC	HP0995 deletion and *difH*WT-*rpsL*-*cat*-*difH*WT cassette insertion between *ureA* and *ureB*	This work
26695^{Str} Δ*xerH*recip	HP0675 deletion and *rpsL-cat* cassette insertion in HP1277	This work
26695^{Str} *xerH*comp	HP0675 deletion and *xerH* complementation in HP1277 locus	This work
26695^{Str} *xerH*comp; *ureAB*::*difH*WT-RC	XerH complemented strain and *difH*WT-*rpsL*-*cat*-*difH*WT cassette insertion between *ureA* and *ureB*	This work
26695^{Str} Δ*ruvC*	HP0877 replaced by *rpsL-cat* cassette	This work
26695^{Str} Δ*recG*	HP1523 replaced by *aphA* replacement	This work
26695^{Str} *recA*::mu-*rpsL-cat*	HP0153 with *rpsL-cat* cassette insertion	This work
26695^{Str} *ftsK*::mu-*rpsL-cat*	HP1090 with *rpsL-cat* cassette insertion	This work
26695^{Str} Δ*xerH*Δ*ruvC*	HP0675 deletion; HP0877 replaced by *rpsL-cat* cassette	This work
26695^{Str} Δ*recGrecA*::mu-*rpsL-cat*	HP1523 replaced by *aphA*; HP0153 with *rpsL-cat* cassette insertion	This work
26695^{Str} Δ*xerH*Δ*recG*	HP0675 deletion; HP1523 replaced by *aphA*	This work
26695^{Str} Δ*ruvC*Δ*recG*	HP0877 replaced by *rpsL-cat*cassette ; HP1523 replaced by *aphA*	This work
26695^{Str} Δ*xerH*Δ*ruvC*Δ*recG*	HP0675 deletion; HP0877 replaced by *rpsL-cat* cassette; HP1523 replaced by *aphA*	This work
X47	Streptomycin resistant	(Kleanthous et al., 2001; Handt et al., 1995)
X47 *xerH*::*rpsL-cat*	HP0675 with *rpsL-cat* cassette insertion	This work
X47 Δ*xerH*	HP0675 deletion	This work
X47 Δ*ruvC*	HP0877 replaced by *rpsL-cat* cassette	This work

Benghezal, M., Dieye, Y., Schwan, C., Sehnal, M., Fulurija, A., and Marshall, B.J. (2009). Synthetic operon. Australian Patent Application No 2009902990

Dailidiene, D., Dailide, G., Kersulyte, D., and Berg, D.E. (2006). Contraselectable streptomycin susceptibility determinant for genetic manipulation and analysis of *Helicobacter pylori*. Appl Environ Microbiol *72*, 5908-5914.

Kleanthous, H., Tibbitts, T.J., Gray, H.L., Myers, G.A., Lee, C.K., Ermak, T.H., and Monath, T.P. (2001). Sterilizing immunity against experimental *Helicobacter pylori* infection is challenge-strain dependent. Vaccine *19*, 4883-4895.

Akopyants, N.S., Eaton, K.A., and Berg, D.E. (1995). Adaptive mutation and cocolonization during *Helicobacter pylori* infection of gnotobiotic piglets. Infect Immun *63*, 116-121.
Handt, L.K., Fox, J.G., Stalis, I.H., Rufo, R., Lee, G., Linn, J., Li, X., and Kleanthous, H. (1995). Characterization of feline *Helicobacter pylori* strains and associated gastritis in a colony of domestic cats. J Clin Microbiol *33*, 2280-2289.
Takeshita, S., Sato, M., Toba, M., Masahashi, W., and Hashimoto-Gotoh, T. (1987). High-copy-number and low-copy-number plasmid vectors for lacZ alpha-complementation and chloramphenicol- or kanamycin-resistance selection. Gene *61*, 63-74.
