# Supplementary material for: Xer Recombinase and Genome Integrity in Helicobacter pylori, a Pathogen without Topoisomerase IV
Source: PLoS One. 2012 Apr 12;7(4):e33310. doi: 10.1371/journal.pone.0033310 (PMC3325230; doi:10.1371/journal.pone.0033310)
Supplement: Table S2 — Oligonucleotide primers used in this study. (PDF) [file pone.0033310.s004.pdf]

Table S2. Oligonucleotide primers used in this study.

Name	Sequence (5'--> 3')
difHF	TATCGAGGATCCATTTAAAAGTTTGAAAAGTGCAGTTTTCATAACTAAATGAGATGC TTTATAACTATGGATTAAACAC
difHR	ACGTTGGATCCTCATTTAGTTATGAAAACTGCACTTTTCAAACTTTTAAATGGATTTT ATTTATTCAGCAAGTCTTG
NondifF	TATCGAGGATCCAGAAGACGGTAGAACCTTGTCTGACTACAACATCCAAAAGGATG CTTTATAACTATGGATTAAACAC
NondifR	ACGTTGGATCCCTTTTGGATGTTGTAGTCAGACAAGGTTCTACCGTCTTCTGGATTT TATTTATTCAGCAAGTCTTG
SEQdifF	GTATTAACAGCTATTAGAGCTAC
SEGdifR	ACGATAGCTTCTTGTAATGCAG
XerHrcat1	GAAAATGATGAGTTGCCCAACAC
XerHrcat2	CCATAGTTATAAAGCATCTGACTCCACTGATGTTTTTAAGCGTG
XerHrcat3	GCTGAATAAATAAAATCCATCAAAGCGCAGGCAATAAGCTC
XerHrcat4	GATGCGGCCAATTTTTCTCATAACAC
XerHrcat5	CATCAGTGGAGTCAGATGCTTTATAACTATGGATTAAACAC
XerHrcat6	GCCTGCGCTTTGATGGATTTTATTTATTCAGCAAGTCTTG
XerHrcat7	CTATCTATGGGTGTATTGATGATG
XerHrcat8	CAAGCCTTGGAGCGTTATTTCTTAG
XerTrcat1	GACCAAATTAAAACAGCGACTTACTC
XerTrcat2	CACACTATAAAGACCTTTCTTTAAGTG
XerTrcat3	GAATAAATAAAATCCATGCTTGGCTTAGTGATGAATATAGAC
XerTrcat4	CACTAAGCCAAGCATGGATTTTATTTATTCAGCAAGTCTTG
XerTrcat5	CTCATGACATGATATTTACTAATAACATG
XerTrcat6	CGCTTTTATATCTAATACCCATAAACAC
XerTrcat7	GGTAGAAAAGAGAGAAAAGGATGCTTTATAACTATGGATTAAACAC
XerTrcat8	GCATCCTTTTCTCTCTTTTCTACCTTTACCTTG
XerHdel1	CCAAAAAAATTCTCCATCAATTTCTTC
XerHdel2	GTGTTTTTAATTTTCTTAAGTTTAGTCATCTATTAAGTGAATG
XerHdel3	GATGACTAAACTTAAGAAAATTAAAAACACCCCCTACTC
XerHdel4	TAGCGGATACTAAACGCTATCATC
XerHdel5	AAGAGAAGTAAGAGATACCTTTGATC
XerHdel6	ACGCTCACAGGGTTTTTTGCTATC
XerTHdel1	CAAAAACGCTTTAAAACGCCTTGTAG
XerTHdel2	CATAACAAACTCGGTGGTTAAAATTGATTAGGTAGTTTAG
XerTHdel3	GTTTTCTTAAACATAATCATAAAGCAAG
XerTHdel4	GATTTGTTTGTCAATGATGCTCAATG
XerTHdel5	CCTAATCAATTTTAACCACCGAGTTTGTTATGTGGCATATTGCTC

XerTHdel6	CCTAGTAGTATTAAGTTTGGAGGAG
UreABF	CTTCTTGTTTCGTCTAACTGCCAAG
UreABR	ATCGCACCAGCTTCAATTTGATC
XerHF	CTTGTGAATTCCATATGAAACACCCCCTAGAAGAATTG
XerHR	GACAAGTCGACAGATCTTTAATTTTCTTCCCAAATGCTCGCC
RuvCrcat1	AACAGCTTGTCTTTGACTTCGTTAC
RuvCrcat2	CATCATCTATTCCTAAAATACGCATCAACCATTC
RuvCrcat3	CTTGCTGAATAAATAACTACCATTTGTAAGCCACTTC
RuvCrcat4	CCTCATAAAATCATCACTTTTGGAG
RuvCrcat5	GATGCGTATTTTAGGAATAGATGATGCTTTATAACTATGGATTAAACAC
RuvCrcat6	CTTACAAATGGTAGTTATTTATTCAGCAAGTCTTGTAATTCATC
RuvCrcat7	TAAGGAGAGACAGCCTGTTAGAC
RuvCrcat8	GATAATTTCATCTCACAATACGCAC
RecGkan1	TTTTAGTCTAGATGTTGTTTTGGTGCGATTGAGAC
RecGkan2	GATCCTCATAGTCTGAATTCCAATGTATTTTAATGCATTTAAATGTTGTGAAG
RecGkan3	CATTGGAATTCAGACTATGAGGATCCTTAAAGGCCGCTAGCGTTTCAC
RecGkan4	AGTTTGTCTAGAAGTTGAGCGCATTCGTTATCGTC
RecAF	GCTATGAGCGACACTAATCAAAG
RecAR	TTGCCCCTACTATCCATCACTTC
FtsKF	ATGAAATCTAAAAAACTTTATTTGG
FtsKR	CTAAAAGTTTTGCAAAATCTCTCTGTTG

a Based on sequence of H. pylori strain 26695 [20]

Function of PCR product
difH -RCAT-*difH* for pHInt_difH-RCAT-difH
nondif -RCAT-*nondif* for pHInt_nondif-RCAT-nondif
Sequencing primer for pHInt_difH-RCAT-difH
Inactivation of *xerH* with *rpsL-cat*
Inactivation of *xerT* with *rpsL-cat*
Complete removal of *xerH*
Complete removal of *xerT*

Screening of *ureAB* locus

xerH complementation

Inactivation of *ruvC* with *rpsL-cat*

Inactivation of *recG* with *alpha* cassette

Inactivation of *recA* with *rpsL-cat*

Inactivation of *ftsK* with *rpsL-cat*
